# Thoracic kyphosis is associated with somatosensory dependence and postural control in adolescent idiopathic scoliosis

**DOI:** 10.1371/journal.pone.0361226

**Published:** 2026-10-23

**Authors:** Wenjun Jiang, Ningnan Zhu, Peng Zhang, Jiahuan Wu, Sheng Wang, Xueliang Wu

**Affiliations:** 1 Suzhou Vocational Health College, Suzhou, China; 2 Suzhou Research Center of Medical School, Suzhou Hospital, Affiliated Hospital of Medical School, Nanjing University, Suzhou, China; University of Tehran, IRAN, ISLAMIC REPUBLIC OF

## Abstract

**Background:**

The contribution of sagittal spinal alignment to sensorimotor function in adolescent idiopathic scoliosis (AIS) remains unclear. We examined whether thoracic kyphosis (TK) is associated with sensory integration and postural balance in patients with AIS, independent of coronal structural misalignment.

**Methods:**

Forty-five AIS patients (Cobb angle ≥ 10°) were consecutively recruited from the outpatient rehabilitation clinic of a university-affiliated hospital (median age 11.0 years, IQR 10.0–13.0; 33 female). There was no selection criterion related to kyphosis. All participants were newly screened and had not yet initiated scoliosis-specific exercise, physiotherapy, or brace treatment. Participants completed the Sensory Organization Test (SOT) and full-spine radiography. Five sensory ratios and four postural control indices were derived from SOT condition-level equilibrium scores (ES). The C4/C1 ratio reflects the ability to maintain postural stability when somatosensory input is disrupted by a sway-referenced support surface (C4) relative to a fixed surface (C1). Associations were examined with Spearman and partial Spearman correlations (adjusting for Cobb angle, age, and BMI) and tertile comparisons with false discovery rate (FDR) correction.

**Findings:**

Forty-two patients had complete data for primary analyses (median Cobb angle 15.3°, IQR 11.2°–20.0°). Greater TK was associated with a higher C4/C1 ratio (Spearman ρ = 0.416, p = 0.006), and this association remained significant after adjustment for Cobb angle, age, and BMI (partial Spearman ρ = 0.475, p = 0.002). Cobb angle was not associated with any sensory ratio (all p > 0.1) but was positively associated with firm-surface equilibrium scores (C1: ρ = 0.525, p < 0.001). The C4/C1 ratio increased monotonically across TK tertiles (Low 0.701 → Mid 0.832 → High 0.861; Kruskal-Wallis H = 8.58, p = 0.014) and was associated with better postural control across all domains (composite ES: ρ = 0.453; variability: ρ = −0.410; adaptation: ρ = 0.433; all p < 0.01).

**Interpretation:**

TK and Cobb angle showed differential associations with postural control: TK was associated with the C4/C1 ratio, whereas Cobb angle was associated with firm-surface stability. The C4/C1 ratio statistically carried the association between TK and postural control via an indirect-only pathway. These findings suggest that sagittal and coronal spinal parameters are differentially associated with postural control, which may inform future research on phenotype-specific assessment strategies.

## Introduction

Adolescent idiopathic scoliosis (AIS) is a three-dimensional spinal curvature affecting 2–4% of adolescents, characterised by lateral curvature (Cobb angle ≥ 10°), axial rotation, and altered sagittal alignment [1–3]. A recent comprehensive review has updated the understanding of its multifactorial aetiology and clinical management [1]. Although clinical assessment and surgical decision-making have traditionally centred on the magnitude of coronal curvature, a growing body of evidence indicates that sagittal plane parameters, particularly thoracic kyphosis (TK), are equally important correlates of functional outcome [4–6].

Postural control deficits are well documented in AIS. Compared with healthy controls, patients with AIS exhibit greater postural sway, altered sensory weighting, and a reduced capacity to resolve sensory conflict [2,3]. Recent work has highlighted sensorimotor integration deficits as a potential aetiological factor in AIS, assessed via diffusion tensor imaging, which has revealed disruptions in both somatosensory afferent and motor efferent pathways in AIS [7].

The Sensory Organization Test (SOT), a standardised protocol comprising six conditions of progressively increasing difficulty, provides a systematic means of quantifying sensory integration. The six conditions are: C1 (eyes open, fixed support, fixed visual surround), C2 (eyes closed, fixed support), C3 (eyes open, fixed support, sway-referenced visual surround), C4 (eyes open, sway-referenced support, fixed visual surround), C5 (eyes closed, sway-referenced support), and C6 (eyes open, sway-referenced support, sway-referenced visual surround). By independently manipulating visual and somatosensory input, the SOT reveals how the central nervous system weights and reweights sensory information to maintain postural stability [4,5]. The SOT, originally developed for astronauts, has evolved substantially with the advent of virtual-reality-based alternatives, yet remains a clinical standard for quantifying sensory contributions to balance [5]. The SOT distinguishes between somatosensory (peripheral proprioception and plantar cutaneous input), visual, and vestibular contributions, though the standard protocol does not fully separate peripheral somatosensory from vestibular function.

The C4/C1 ratio (somatosensory dependence ratio) is of particular interest because it isolates the effect of disrupting somatosensory input on postural stability: C4 introduces a sway-referenced support surface that reduces the reliability of proprioceptive and plantar cutaneous feedback while visual information remains available. A higher C4/C1 ratio indicates relatively preserved performance when somatosensory input is disrupted, which may reflect effective use of remaining sensory information (visual and/or vestibular) to compensate for somatosensory perturbation. This ratio has been used in previous studies to quantify the somatosensory contribution to postural control [6,8].

A key unresolved question is whether postural control deficits in AIS are associated primarily with coronal curvature (Cobb angle), with sagittal alignment (TK), or with both. From a biomechanical standpoint, thoracic kyphosis may be especially consequential: the posterior shift of the trunk centre of mass that accompanies increasing TK alters the geometry of the postural control system, potentially modifying reliance on somatosensory feedback from the lower extremities and trunk [9,10]. The clinical evidence, however, remains inconsistent, with some studies linking Cobb angle to balance deficits [11,12] and others finding no such association [13,14].

Disentangling the contributions of TK and Cobb angle to postural control is methodologically challenging. First, TK and Cobb angle are negatively correlated in AIS (thoracic hypokyphosis accompanies curve progression [15–17]), so their associations with postural control may be confounded in unadjusted analyses. Second, sensory integration is influenced by multiple factors; age, skeletal maturity, and curve type may each influence SOT performance independent of spinal alignment. Third, the standard practice of applying a Cobb ≥ 10° diagnostic cutoff excludes patients with minor coronal asymmetry, potentially truncating the range of both Cobb and TK values and obscuring gradient relationships.

The present study had three specific aims: (1) to determine whether TK is independently associated with sensory integration patterns in AIS, after accounting for coronal curvature; (2) to characterise the monotonic gradient between TK and the C4/C1 ratio across TK levels; and (3) to test whether the C4/C1 ratio statistically carries the association between TK and postural control performance (i.e., an indirect association pathway), consistent with the hypothesis that sagittal alignment may relate to postural control through sensory integration mechanisms.

We hypothesised that greater TK would be associated with a higher C4/C1 ratio, reflecting relatively preserved postural stability under somatosensory perturbation, and that this association would be independent of Cobb angle.

## Materials and methods

### Participants

Sixty-one patients with AIS were prospectively and consecutively recruited from 7 September 2025–30 June 2026 at the outpatient rehabilitation clinic of the Suzhou Research Center of Medical School, Suzhou Hospital, Affiliated Hospital of Medical School, Nanjing University (Suzhou Science & Technology Town Hospital), Suzhou, China. Inclusion criteria were: (1) radiographic diagnosis of AIS with Cobb angle ≥ 10°; (2) age 8–18 years; (3) no prior spinal surgery; and (4) ability to complete the SOT protocol independently. There was no selection criterion related to kyphosis. All participants were newly screened and had not yet initiated scoliosis-specific exercise, physiotherapy, or brace treatment at the time of assessment.

Exclusion criteria were assessed through clinical examination and medical history review by a rehabilitation physician: neurological disorders, vestibular pathology, lower extremity injury within the preceding six months, and inability to stand unassisted for ≥ 20 seconds. After excluding 12 patients with Cobb < 10° (not meeting the SRS diagnostic threshold [18]) and four patients with gait abnormalities that precluded standardised assessment, 45 patients (33 female; median age 11.0 years, IQR 10.0–13.0, range 8–18) were included in the final analysis. Eight patients (17.8%) were aged 8–9 years, falling within the juvenile idiopathic scoliosis (JIS) range by strict SRS classification. These patients were retained in the primary analysis for the following reasons: (1) the 2024 Chinese AIS rehabilitation guideline recommends scoliosis screening at ages 8–18 years [19], reflecting that the juvenile–adolescent boundary is not rigidly enforced in clinical practice; (2) a prior study demonstrated no significant difference in end-of-growth outcomes between JIS and AIS patients treated after age 10 [20]; and (3) sensitivity analyses excluding these patients confirmed the robustness of the primary findings (see the Robustness checks section and S3 Table in S1 File).

The study was approved by the Institutional Review Board of Suzhou Hospital, Affiliated Hospital of Medical School, Nanjing University (Suzhou Science & Technology Town Hospital) (Approval No. IRB2025076), and written informed consent was obtained from all participants and their guardians.

### Radiographic assessment

Full-length standing posteroanterior and lateral spine radiographs were obtained using a standardised protocol. Radiographs were acquired with participants standing in a relaxed, upright position with arms folded across the chest at approximately 45° to the horizontal plane, feet shoulder-width apart, and gaze directed forward. The following parameters were measured by a single experienced rehabilitation physician using Surgimap software (version 2.3.2.1, Nemaris Inc., USA): major Cobb angle, thoracic kyphosis (TK; T5–T12), lumbar lordosis (LL; L1–S1), C7–central sacral vertical line (C7-CSVL, measured in the frontal (coronal) plane), and apical vertebral translation (AVT, measured for the major curve). Curve direction (left-convex vs. right-convex) and curve type (single thoracic, thoracolumbar/lumbar, double major) were recorded. Skeletal maturity was assessed using the Risse plus (Risser+) classification, coded as an ordinal scale: 0− = 1, 0+ = 2, 1 = 3, 2 = 4, 3 = 5, 3/4 = 6, 4 = 7, 5 = 8. Five participants with unrecorded Risser stage were coded as 0. Intra-rater reliability was assessed by re-measuring 15 randomly selected radiographs after a two-week interval; intraclass correlation coefficients (ICC_3,1_) exceeded 0.95 for TK and 0.97 for Cobb angle.

### Sensory Organization Test (SOT)

Postural control was assessed using the standardised six-condition SOT protocol [6,21] on a computerised dynamic posturography platform (NeuroCom® SMART Balance Master, NeuroCom International, Clackamas, OR, USA). SOT testing and radiography were performed on the same day, with the SOT administered first to avoid fatigue effects from prolonged standing during radiographic positioning. As all participants were pre-treatment, no braces were worn during SOT testing.

Participants stood barefoot with feet positioned according to the manufacturer’s guidelines and were instructed to maintain an upright posture throughout each 20-second trial. The six conditions were administered in the standard fixed order (C1 through C6), with each condition performed in triplicate:

- **C1:** Eyes open, fixed support surface, fixed visual surround
- **C2:** Eyes closed, fixed support surface
- **C3:** Eyes open, fixed support surface, sway-referenced visual surround (visual conflict)
- **C4:** Eyes open, sway-referenced support surface, fixed visual surround
- **C5:** Eyes closed, sway-referenced support surface
- **C6:** Eyes open, sway-referenced support surface, sway-referenced visual surround

The equilibrium score (ES) for each trial was computed as:


$$\begin{array}{c} \text{ES}=\frac{{\text{1}\text{2}.\text{5}}^{\circ }-\theta_{\text{max}}}{{\text{1}\text{2}.\text{5}}^{\circ }}\times \text{1}\text{0}\text{0} \end{array}$$

where θ_max is the maximum anteroposterior sway angle during the trial. The SOT equilibrium score measures sway in the anteroposterior (sagittal) plane, as this is the primary plane of postural control during quiet stance [6,21]. Condition-level scores were averaged across the three trials.

When a participant experienced a fall during a trial (loss of balance requiring assistance), the equilibrium score for that trial was recorded as 0, consistent with the standard SOT scoring convention [6,21]. One participant (P29) experienced falls on all three C6 trials; this participant’s C6 equilibrium score was 0 and was included in all analyses. The mean equilibrium score (MeanES, the mean of C1–C6 condition-level ES values; also referred to as the composite equilibrium score) was computed.

### Derived sensory and postural control indices

**Sensory dependence ratios.** Five sensory dependence ratios were computed from condition-level equilibrium scores following established convention [6,8]:

- **Visual dependence ratio (C2/C1):** Higher values indicate greater reliance on visual input.
- **Somatosensory dependence ratio (C4/C1):** Higher values indicate relatively preserved performance when somatosensory input is disrupted by the sway-referenced support surface. Under the standard SOT convention, this ratio reflects the ability to maintain postural stability when proprioceptive and plantar cutaneous feedback are rendered unreliable while visual and vestibular information remain available. A higher C4/C1 ratio does not necessarily reflect better somatosensory utilisation per se; it may reflect effective compensatory reliance on visual and/or vestibular inputs.
- **Vestibular dependence ratio (C5/C1):** Higher values indicate better vestibular function.
- **Visual preference ratio (C3/C1):** Higher values indicate greater reliance on accurate visual input.
- **Somatosensory/visual preference ratio (C4/C2):** Higher values indicate a relative preference for somatosensory over visual cues when both are available.

**Note on SOT sensory ratio nomenclature.** In this study, sensory dependence ratios are defined by the sensory modality that is challenged between the two conditions being compared: C4/C1 is termed the somatosensory dependence ratio because C4 introduces a sway-referenced support surface that disrupts somatosensory input relative to C1; C2/C1 is termed the visual dependence ratio because C2 removes visual input relative to C1. This functional labelling differs from the manufacturer’s convention (SOM = C2/C1, VIS = C4/C1), which names ratios by the sensory system under test rather than by the modality being challenged. All sensory dependence ratios were computed directly from condition-level equilibrium scores (C1–C6 ES) rather than from the pre-labelled NeuroCom output values, ensuring transparent and reproducible ratio derivation.

**Postural control composite indices** were derived to reduce dimensionality across the six conditions:

- **Mean equilibrium score (MeanES):** The mean of C1 through C6 condition-level ES values. Higher values indicate better overall postural control. This is the standard SOT composite score.
- **Postural reserve deficit (PostRes):** Computed as the residual variance from a participant-level ordinary least squares regression of the six condition-level equilibrium scores on condition number (ES ~ condition_number). Higher values indicate greater deviation from the expected linear decline across conditions, representing a larger postural control deficit.
- **Response variability (CV):** Coefficient of variation of the six condition-level mean equilibrium scores (SD/mean), capturing the magnitude of condition-to-condition fluctuation. Calculated using the mean of the three trials per condition.
- **Adaptation slope (Slope):** The regression coefficient from the participant-level linear regression of ES on condition number (C1 → C6), where a more negative slope indicates greater sensitivity to increasing sensory challenge. Note: this metric assumes approximately equal-interval difficulty steps across SOT conditions (see Limitations).

References for the measurement properties of the SOT, including test-retest reliability of the composite score and sensory ratios, are provided in the published literature [6,8,21].

### Statistical analysis

Normality was assessed using the Shapiro-Wilk test. Given non-normal distributions for several key variables, non-parametric methods were used throughout.

**Primary (prespecified) analyses.** Three analyses were specified a priori: (1) Spearman rank correlation between TK and the C4/C1 ratio; (2) partial Spearman correlation between TK and the C4/C1 ratio, controlling for Cobb angle, age, and BMI jointly [22]; and (3) Kruskal-Wallis test of the C4/C1 ratio across TK tertiles. Covariates were selected for their known or plausible influence on postural control: Cobb angle (coronal curvature severity), age (developmental maturation), and BMI (body habitus effects on postural control).

**Secondary/exploratory analyses.** Secondary analyses included: (1) Spearman correlations between spinal parameters (TK, Cobb, LL) and all sensory ratios and SOT condition scores; (2) Spearman correlations between the C4/C1 ratio and all postural control indices; (3) formal mediation testing following the Baron & Kenny [23] framework: (a) TK → C4/C1; (b) C4/C1 → MeanES (controlling for TK); (c) TK → MeanES (total association); and (c′) TK → MeanES (controlling for C4/C1, direct association). The Sobel test was used to evaluate the statistical significance of the indirect (a × b) pathway. All secondary analyses are explicitly labeled as exploratory.

**Tertile analysis.** For descriptive characterisation of the gradient relationship, participants were stratified into TK tertiles (Low, Mid, High). Group differences in sensory dependence and postural control indices were assessed using the Kruskal-Wallis test with post-hoc Mann-Whitney U tests (Holm-Bonferroni corrected). Tertile boundaries are data-derived and should not be interpreted as clinically meaningful thresholds.

**Multiple testing correction.** False discovery rate (FDR) correction was applied within each analysis family to control for multiple comparisons [24]: (a) the five sensory ratios (5 tests); (b) the six SOT condition scores (6 tests); (c) the seven tertile group comparisons (7 tests); and (d) the four postural control indices (4 tests). Statistical significance was set at α = 0.05 (two-tailed). All analyses were performed in Python 3.12 using SciPy (v1.11) and pandas (v2.1).

## Results

### Missing data

Of the 45 enrolled patients, 42 had complete radiographic and SOT data for all primary analyses. Three patients had incomplete SOT data due to equipment malfunction during one testing session (one patient missing C5 and C6 trials; two patients missing C6 trials only). One additional subject was excluded from partial correlation models due to missing BMI (weight measurement inadvertently omitted). Missingness was not significantly associated with any demographic or clinical variable (age, sex, Cobb angle, TK; all p > 0.30). Sample sizes are noted for each analysis throughout.

### Participant characteristics

Demographic and radiographic characteristics are summarised in Table 1. The cohort (N = 42 with complete data) was predominantly female (73.8%) with mild-to-moderate scoliosis (Cobb range 10.0°–34.7°). Curve types were: single thoracic (Lenke type 1), n = 9 (21.4%); thoracolumbar/lumbar (Lenke type 5), n = 31 (73.8%); and double major (Lenke types 2 and 3), n = 2 (4.8%). Thoracic kyphosis showed substantial inter-individual variability (range 2.0°–53.2°, coefficient of variation = 37.8%), exceeding that of lumbar lordosis (CV = 24.1%).

**Table 1:** Participant demographics and radiographic characteristics (N = 42).

Parameter	Mean ± SD	Median (IQR)	Range
Age (years)	11.6 ± 2.2	11.0 (10.0–13.0)	8.0–18.0
Sex (F/M)	31 / 11	—	—
Height (cm)	154.1 ± 13.7	154.5 (143.0–164.0)	125.0–182.0
Weight (kg)	42.3 ± 13.5	40.0 (31.0–52.0)	21.5–78.0
BMI (kg/m²)	17.3 ± 3.1	16.8 (15.0–19.0)	11.4–23.8
Risser grade (Risser + , ordinal)	3.7 ± 2.7	3.0 (2.0–6.8)	0–8
Major Cobb angle (°)	16.2 ± 6.1	15.3 (11.2–20.0)	10.0–34.7
Thoracic kyphosis (°)	28.7 ± 10.9	28.5 (21.0–36.0)	2.0–53.2
Lumbar lordosis (°)	50.2 ± 12.1	50.5 (42.0–60.0)	25.0–71.0
C7-CSVL (frontal, mm)	14.0 ± 8.4	13.0 (8.0–20.0)	0.0–33.0
AVT (major curve, mm)	17.6 ± 8.8	17.0 (10.0–23.0)	0.0–34.7
Curve direction (L/R convex)	26 / 16	—	—
Single thoracic, n (%)	9 (21.4%)	—	—
Thoracolumbar/lumbar, n (%)	31 (73.8%)	—	—
Double major, n (%)	2 (4.8%)	—	—

All participants were newly screened and had not yet initiated scoliosis-specific exercise, physiotherapy, or brace treatment at the time of assessment.

### SOT performance and sensory dependence patterns

SOT equilibrium scores across conditions followed the expected decreasing pattern with increasing sensory challenge (Table 2). The mean composite equilibrium score (MeanES) was 71.4 ± 8.1 (median 70.8, IQR 66.0–78.0), consistent with published AIS norms [25,26]. Individual variability was greatest in the most challenging conditions (C5: CV = 29.9%; C6: CV = 36.9%).

**Table 2:** SOT Condition-Level Equilibrium Scores, Derived Sensory Ratios, and Postural Control Indices (N = 42).

Measure	Mean ± SD	Median (IQR)	Range
C1 (Eyes open, fixed support, fixed visual surround)	89.1 ± 5.9	90.4 (86.0–93.0)	72.0–97.0
C2 (Eyes closed, fixed support)	87.0 ± 5.3	88.3 (83.0–91.0)	70.7–95.0
C3 (Eyes open, fixed support, sway-referenced visual surround)	82.2 ± 8.3	83.3 (78.0–88.0)	59.0–95.7
C4 (Eyes open, sway-referenced support, fixed visual surround)	70.7 ± 13.8	72.2 (62.0–81.0)	43.3–92.0
C5 (Eyes closed, sway-referenced support)	53.1 ± 15.9	52.5 (42.0–65.0)	20.3–82.0
C6 (Eyes open, sway-referenced support, sway-referenced visual surround)	46.6 ± 17.2	48.0 (37.0–60.0)	0.0–81.7
Visual dependence (C2/C1)	0.977 ± 0.041	0.971 (0.950–0.997)	0.907–1.127
Somatosensory dependence (C4/C1)	0.795 ± 0.153	0.840 (0.695–0.911)	0.280–0.985
Vestibular dependence (C5/C1)	0.593 ± 0.166	0.588 (0.470–0.727)	0.246–0.888
Visual preference (C3/C1)	0.923 ± 0.076	0.931 (0.878–0.971)	0.683–1.041
Somatosensory/visual preference (C4/C2)	0.813 ± 0.153	0.867 (0.704–0.919)	0.306–1.038
MeanES (Composite ES)	71.4 ± 8.1	70.8 (66.0–78.0)	53.8–88.2
PostRes (deficit)	38.2 ± 13.3	36.6 (28.0–46.0)	14.3–70.8
CV (variability)	0.279 ± 0.120	0.260 (0.190–0.340)	0.075–0.560
Slope (adaptation)	−9.31 ± 3.30	−8.88 (−11.5 to −7	.00) −17.9 to −3.45

*Note:* C1–C6 values are condition-level equilibrium scores (ES). PostRes, CV, and Slope are derived postural control indices as defined in Section 2.4. FALL trials were scored as 0 per standard SOT convention.

### Primary analysis: TK is associated with the C4/C1 ratio independently of Cobb angle

*Direction note:* A higher C4/C1 ratio indicates relatively preserved performance on the sway-referenced platform, i.e., less degradation when somatosensory input is disrupted. This reflects the ability to maintain postural stability using remaining sensory information (visual and/or vestibular). Alternative interpretations are considered in the Discussion.

Thoracic kyphosis was significantly correlated with the C4/C1 ratio (somatosensory dependence: Spearman ρ = 0.416, p = 0.006, n = 42; somatosensory/visual preference (C4/C2): ρ = 0.428, p = 0.005). Patients with greater TK showed less degradation when transitioning from a firm to a sway-referenced surface. This association remained significant after controlling for Cobb angle, age, and BMI jointly (partial Spearman ρ = 0.475, p = 0.002, n = 41; Table 3, Fig 1).

**Table 3:** Spearman correlations: Spinal parameters vs. sensory dependence ratios.

Spinal Parameter	C2/C1	C4/C1	C5/C1	C3/C1	C4/C2
**TK**	−0.050	**0.416**	0.156	−0.125	**0.428**
	p = 0.753	**p = 0.006**	p = 0.325	p = 0.430	**p = 0.005**
**Cobb**	0.103	0.044	0.241	0.230	0.058
	p = 0.517	p = 0.784	p = 0.124	p = 0.143	p = 0.715
**LL**	−0.208	0.312	−0.087	0.022	0.386
	p = 0.187	p = 0.044	p = 0.583	p = 0.890	p = 0.012

Values are Spearman ρ, n = 42. Bold = significant after FDR correction across all 15 comparisons (5 ratios × 3 parameters).

**Fig 1:**
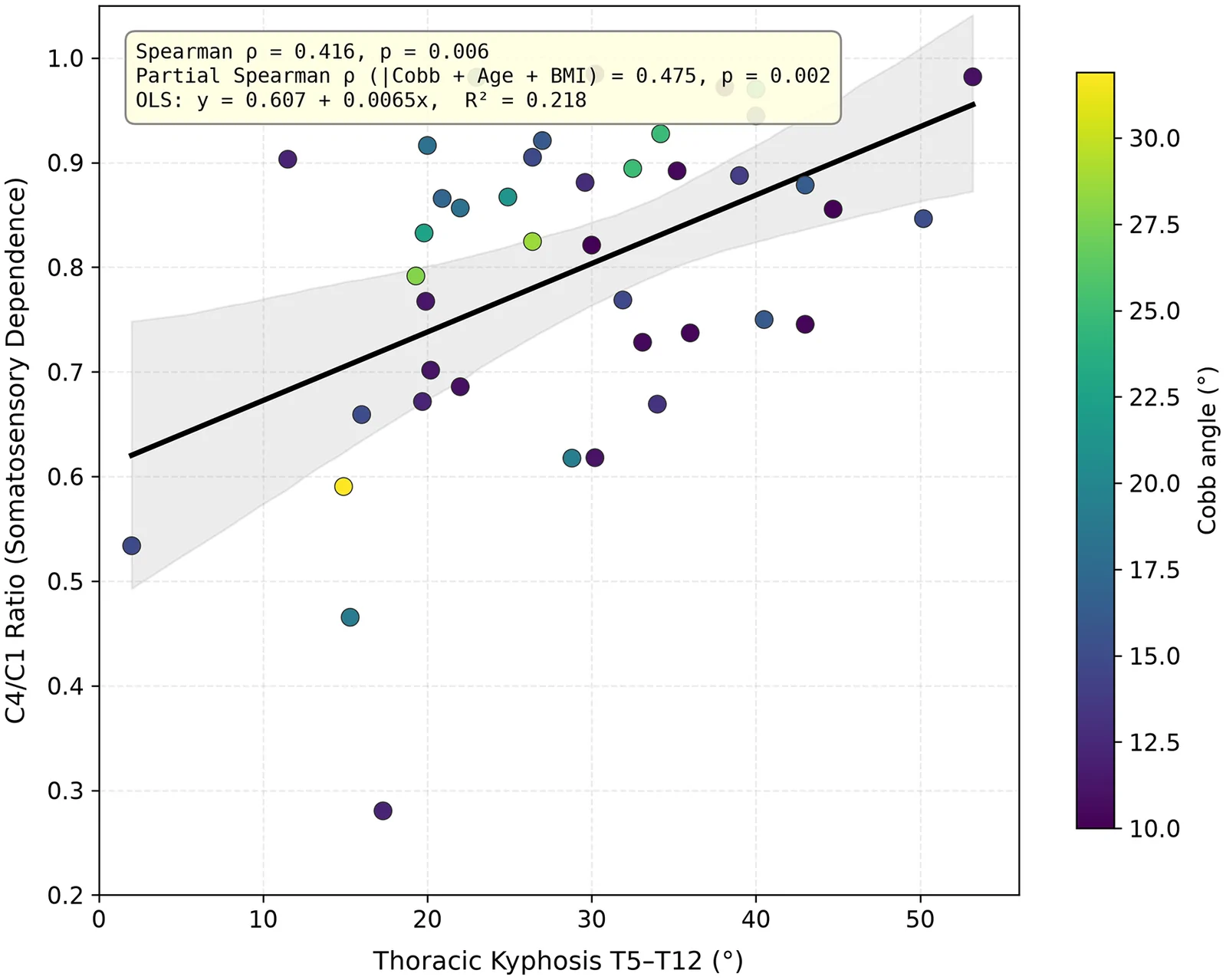
Scatterplot of thoracic kyphosis versus the C4/C1 ratio with an ordinary least squares (OLS) linear fit (solid line) and 95% bootstrap confidence band (grey shading). N = 42. Points are coloured by Cobb angle (continuous viridis scale) to illustrate that the TK–C4/C1 association is independent of coronal curvature severity. Inset: Spearman ρ = 0.416, p = 0.006; partial Spearman ρ (|Cobb + Age + BMI) = 0.475, p = 0.002.

By contrast, Cobb angle showed no significant association with any sensory dependence measure (C4/C1: ρ = 0.044, p = 0.784; C2/C1: ρ = 0.103, p = 0.517; C5/C1: ρ = 0.241, p = 0.124; C3/C1: ρ = 0.230, p = 0.143; C4/C2: ρ = 0.058, p = 0.715). Lumbar lordosis showed a weaker association with the C4/C2 ratio (ρ = 0.386, p = 0.012) but was not significantly associated with the C4/C1 ratio after controlling for TK.

### Monotonic gradient: TK tertile analysis

When stratified by percentile-based TK tertiles, the C4/C1 ratio exhibited a clear monotonic gradient (Table 4). The Low TK group (n = 14; TK ≤ 22°) showed the lowest C4/C1 ratio (0.701 ± 0.178), the Mid TK group (n = 14; TK > 22° and < 34°) showed intermediate values (0.832 ± 0.120), and the High TK group (n = 14; TK ≥ 34°) showed the highest values (0.861 ± 0.100). This gradient was statistically significant (Kruskal-Wallis H = 8.58, p = 0.014). Tertile boundaries are data-derived and should not be interpreted as clinically meaningful thresholds.

**Table 4:** Sensory dependence and postural control by TK tertile.

	Low TK (n = 14)	Mid TK (n = 14)	High TK (n = 14) K	-W H p	
TK range (°)	≤ 22.0	> 22.0, < 34.0	≥ 34.0	—	—
**Sensory dependence ratios**					
Somatosensory dependence (C4/C1)	0.690 ± 0.173	0.834 ± 0.111	0.861 ± 0.096	8.58	**0.014**
Som/Vis preference (C4/C2)	0.706 ± 0.174	0.847 ± 0.101	0.886 ± 0.098	8.64	**0.013**
Visual dependence (C2/C1)	0.975 ± 0.034	0.984 ± 0.051	0.972 ± 0.040	0.24	0.885
Vestibular dependence (C5/C1)	0.532 ± 0.168	0.609 ± 0.151	0.638 ± 0.152	2.74	0.255
**Postural control indices**					
CV (variability)	0.358 ± 0.133	0.256 ± 0.097	0.228 ± 0.068	8.42	**0.015**
Slope (adaptation)	−11.47 ± 3.57	−8.80 ± 2.76	−7.65 ± 1.96	9.43	**0.009**
PostRes (deficit)	46.14 ± 14.30	36.42 ± 11.68	32.04 ± 8.37	7.92	**0.019**

Bold p-values are significant after FDR correction across all seven group comparisons. Note: PostRes is coded such that lower values indicate better postural control (see Section 2.4). Sensory dependence ratios and postural control indices are grouped separately for clarity.

Post-hoc pairwise comparisons showed that the Low TK group differed significantly from both the Mid TK (p = 0.040) and High TK (p = 0.014) groups, whilst Mid and High TK did not differ significantly (p = 0.310). These tertile comparisons are descriptive and should be interpreted with caution given the small group sizes (n = 14 per group) and potential influence of individual observations.

Postural control indices also varied systematically across TK tertiles (Table 4). With increasing TK, the adaptation slope became progressively less negative from Low TK (−11.47 ± 3.57) to High TK (−7.65 ± 1.96; H = 9.43, p = 0.009), and response variability decreased from 0.358 ± 0.133 (Low) to 0.228 ± 0.068 (High; H = 8.42, p = 0.015). The postural reserve deficit (PostRes) was also significant after FDR correction (Low: 46.14 ± 14.30; High: 32.04 ± 8.37; H = 7.92, p = 0.019). Visual dependence and vestibular dependence did not differ across TK tertiles (p > 0.20), confirming the sensory-modality specificity of the TK effect (Fig 2).

**Fig 2:**
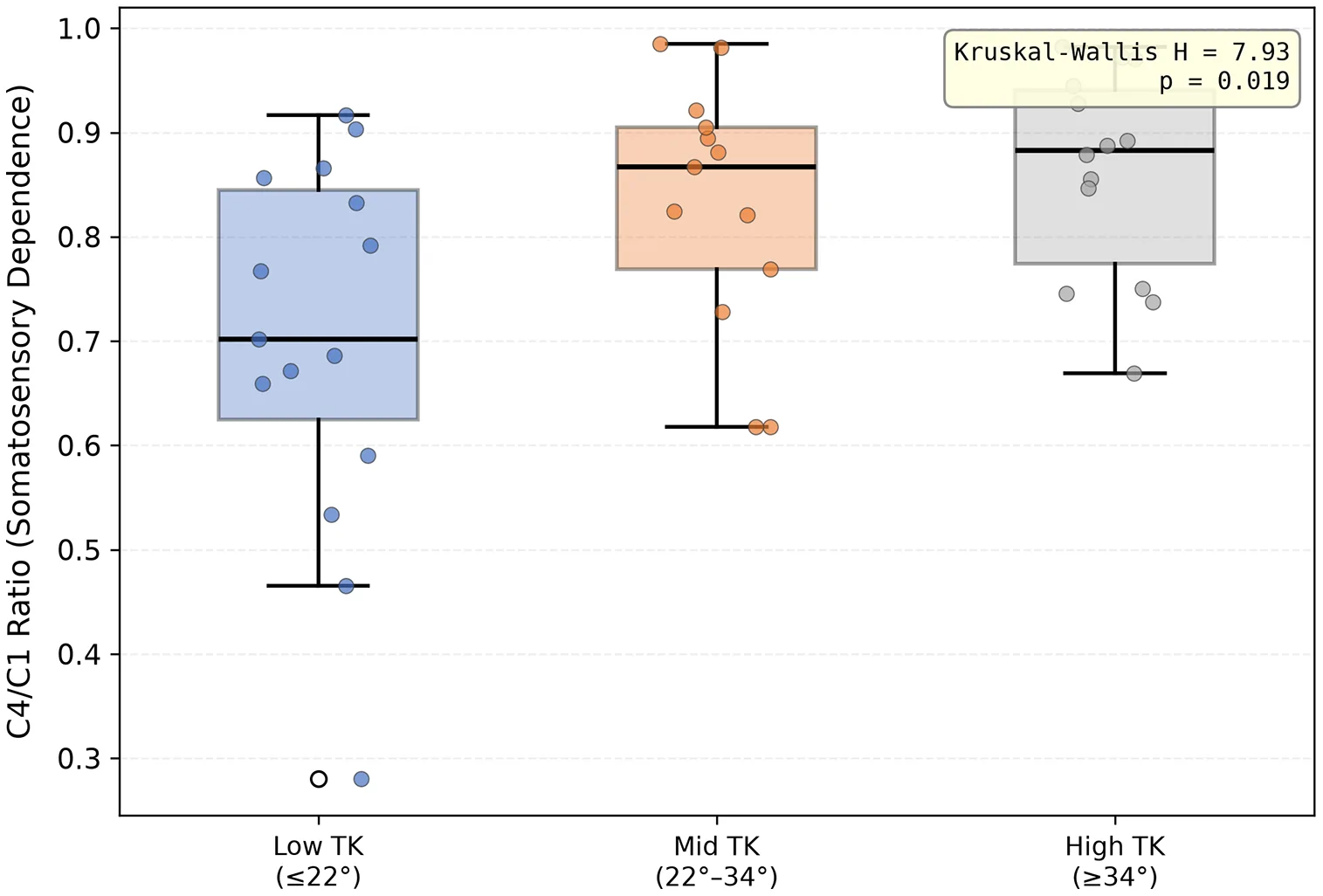
C4/C1 ratio by thoracic kyphosis tertile. Boxes show median and IQR; whiskers extend to 1.5 × IQR; individual points represent jittered subject data. Kruskal-Wallis H = 8.58, p = 0.014. Brackets indicate Mann-Whitney post-hoc pairwise comparisons (Holm-Bonferroni corrected). Low TK (≤22°): n = 14; Mid TK (>22°, < 34°): n = 14; High TK (≥34°): n = 14. Tertile boundaries are data-derived and should not be interpreted as clinically meaningful thresholds. Note that the tertile comparisons are descriptive and may be influenced by individual observations given the small group sizes.

### Sensory–postural association pathway (exploratory)

The C4/C1 ratio was significantly correlated with all postural control indices (Table 5). Higher C4/C1 (less degradation on the sway-referenced surface) was associated with better mean equilibrium (MeanES: ρ = 0.453, p = 0.003), lower postural reserve deficit (PostRes: ρ = −0.368, p = 0.017), reduced response variability (CV: ρ = −0.410, p = 0.007), and a less negative adaptation slope (Slope: ρ = 0.433, p = 0.004). The C4/C1–MeanES correlation was not driven by shared C1 variance; partialling out C1 strengthened the association (partial Spearman ρ = 0.602, p < 0.001), suggesting that the C4/C1 ratio likely reflects between-condition sensory reweighting rather than being solely a mathematical artifact of the ratio construction. Furthermore, C4/C1 was not associated with C1 baseline values (C4/C1 ↔ C1: ρ = −0.031, p = 0.844), weakening the alternative interpretation that high C4/C1 merely reflects floor effects on firm surfaces.

**Table 5:** C4/C1 ratio and postural control.

Postural Control Index	ρ with C4/C1 p	I	nterpretation
Mean Equilibrium (MeanES)	0.453	0.003 H	igher C4/C1 → better overall balance
Postural Reserve Deficit (PostRes)	−0.368	0.017 H	igher C4/C1 → lower deficit (better)
Response Variability (CV)	−0.410	0.007 H	igher C4/C1 → less variability
Adaptation Slope (Slope)	0.433	0.004 H	igher C4/C1 → flatter slope

All *p*-values significant after FDR correction. MeanES: higher = better. PostRes: higher = greater deficit (worse).

**Exploratory mediation analysis** (Baron & Kenny, 1986; Fig 3) showed a significant indirect statistical pathway: TK → C4/C1 (a-path: ρ = 0.416, p = 0.006) and C4/C1 → MeanES (b-path, controlling for TK: ρ = 0.412, p = 0.007), with a significant Sobel test for the indirect association (z = 2.49, p = 0.013). However, the total association of TK with MeanES was not significant (c-path: ρ = 0.21, p = 0.18), and the direct association was near zero after controlling for C4/C1 (c′-path: ρ = 0.03, p = 0.87). This pattern — a significant indirect path without a significant total association — is consistent with *indirect-only mediation* [27], in which the C4/C1 ratio carries the association between TK and postural control, but TK itself does not show a direct association with postural performance. This pattern can occur when the indirect and direct associations have opposing signs, resulting in a cancelled total association. We emphasise that this interpretation is statistical and does not imply causal mediation; temporal precedence cannot be established with cross-sectional data.

**Fig 3:**
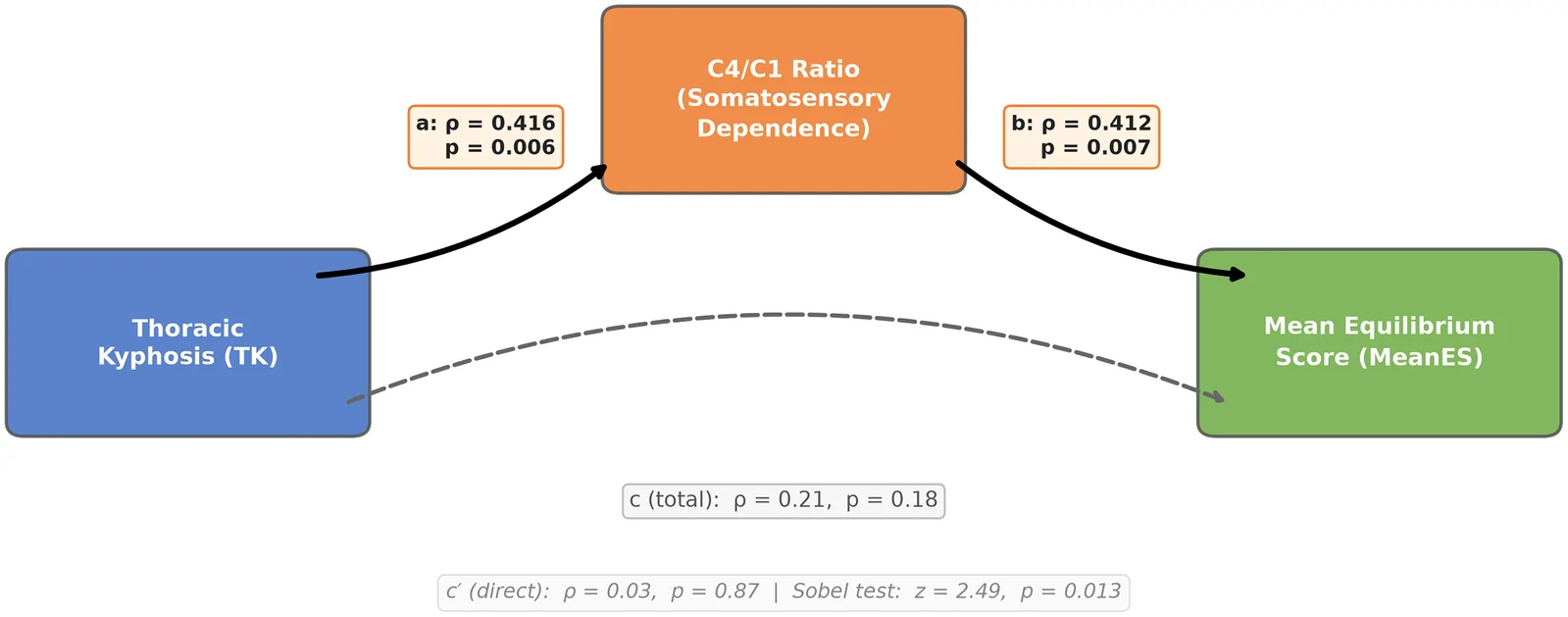
Exploratory mediation analysis framework (Baron & Kenny, 1986). Path a: TK → C4/C1 (Spearman ρ = 0.416, p = 0.006). Path b: C4/C1 → MeanES, controlling for TK (partial Spearman ρ = 0.412, p = 0.007). Path c (total association): TK → MeanES (ρ = 0.21, p = 0.18). Path c′ (direct association): TK → MeanES, controlling for C4/C1 (ρ = 0.03, p = 0.87). Sobel test for indirect association (a × b): z = 2.49, p = 0.013. This analysis is exploratory and statistical; causal mediation cannot be inferred from cross-sectional data.

**Sensitivity analysis for mathematical coupling.** To address the concern that the C4/C1 ratio and MeanES share constituent SOT variables, we repeated the mediation analysis using an outcome that excludes C4: the mean of C1, C2, C3, C5, and C6 (MeanES_noC4). The indirect pathway was attenuated (Sobel z = 1.83, p = 0.067), consistent with the expected reduction in statistical power when C4 is removed from the outcome variable. The direction of the indirect association remained positive, suggesting that the mediation finding is not solely attributable to mathematical coupling, though the attenuation to marginal significance indicates that shared C4 variance contributes to the strength of the primary mediation result.

### Robustness checks (exploratory)

Sensitivity analyses confirmed the primary findings.

**Age criterion sensitivity.** To address the concern that 8 patients were in the juvenile age range (8–9 years), all primary analyses were repeated after excluding these patients. The bivariate TK–C4/C1 correlation was attenuated but remained close to the significance threshold (ρ = 0.329, p = 0.058, n = 34), while the partial Spearman correlation controlling for Cobb angle, age, and BMI remained significant (ρ = 0.368, p = 0.035, n = 33). The complementary pattern of Cobb–TK associations with SOT conditions was preserved: Cobb angle remained associated with firm-surface conditions (C1: ρ = 0.563, p = 0.001; C2: ρ = 0.671, p < 0.001; C3: ρ = 0.570, p < 0.001), and TK remained associated with the most challenging condition (C6: ρ = 0.365, p = 0.034). The C4/C1 ratio remained significantly associated with most postural control indices (MeanES: ρ = 0.500, p = 0.003; CV: ρ = −0.369, p = 0.032; Slope: ρ = 0.368, p = 0.032), though the C4/C1–PostRes association was attenuated (ρ = −0.317, p = 0.068). The tertile and mediation analyses lost statistical significance due to the reduced sample size, but the direction and magnitude of associations remained consistent with the primary analysis. Full results are reported in S4 Table in S1 File. These findings indicate that the primary results are not driven by the inclusion of juvenile-aged patients.

**Hypokyphosis exclusion.** Excluding six patients with hypokyphosis (TK < 15°) did not alter the TK–C4/C1 association (ρ = 0.382, p = 0.019, n = 36). The association was independent of skeletal maturity (Risser interaction p = 0.634) and curve direction (left vs. right: p = 0.472). When TK and LL were jointly examined, TK remained the dominant correlate of the C4/C1 ratio (partial Spearman ρ = 0.420, p = 0.006), whereas LL became non-significant after controlling for TK (partial ρ = 0.280, p = 0.076).

To address the concern that the primary TK–C4/C1 association might be driven by patients with abnormally elevated thoracic kyphosis (potentially representing non-idiopathic pathology), the analysis was repeated after sequentially excluding patients at the upper end of the TK distribution. The effect size remained stable: excluding patients with TK ≥ 40° (n = 37, ρ = 0.319, p = 0.054) and those with TK ≥ 34° (n = 29, ρ = 0.330, p = 0.075) yielded Spearman coefficients comparable to the full-sample estimate, indicating that the association is not driven solely by patients at the extreme upper end of the TK distribution, although statistical significance is attenuated by the corresponding loss of sample size.

## Discussion

This study demonstrates a differential association pattern in how sagittal and coronal spinal parameters relate to postural control in AIS. Thoracic kyphosis is selectively associated with the C4/C1 ratio, whilst Cobb angle is selectively associated with firm-surface equilibrium scores. The C4/C1 ratio is in turn associated with better overall postural control, and statistically carries the TK–postural control association via an indirect-only pathway. Three principal findings emerge: (1) TK is independently associated with the C4/C1 ratio, with the association remaining significant after covariate adjustment; (2) this association exhibits a clear monotonic gradient across TK tertiles; and (3) the C4/C1 ratio is associated with better postural control across multiple domains.

### Differential associations of sagittal and coronal parameters

The most striking finding is the differential pattern of associations between TK and Cobb angle in their relationship to postural control. Whilst TK was robustly associated with the C4/C1 ratio (all Cobb–ratio correlations p > 0.1), Cobb angle was associated with equilibrium scores on firm-surface conditions (C1: ρ = 0.543; C2: ρ = 0.606; C3: ρ = 0.545; all p < 0.001), with higher Cobb counterintuitively associated with *better* firm-surface performance. This pattern — Cobb associated with baseline postural stability, TK associated with performance under sensory perturbation — suggests that coronal and sagittal parameters are associated with postural control through distinct pathways.

The positive Cobb–firm-surface association warrants careful consideration, as it is the strongest set of correlations in the study. Several explanations are plausible. First, chronic postural perturbation from progressive curvature may drive long-term compensatory adaptation of the postural control system, a form of “training effect” in which patients with larger curves develop more robust balance strategies on stable surfaces. Treatment history is unlikely to confound this association, as all participants were newly screened and had not yet initiated any scoliosis-specific intervention. Second, increased trunk rigidity associated with curve progression may mechanically constrain sway amplitude, artefactually elevating equilibrium scores on firm surfaces. Third, Cobb angle is correlated with maturation (Risser stage, age), and the firm-surface association may partly reflect developmental improvements in postural control rather than curve severity per se. However, the Cobb–C1 association remained significant after controlling for age and Risser stage (partial Spearman ρ = 0.41, p = 0.008), arguing against a pure maturational confound. Fourth, the SOT equilibrium score measures anteroposterior (sagittal plane) sway, which may be more sensitive to sagittal plane changes (TK) than to coronal plane changes (Cobb angle), potentially explaining why TK, rather than Cobb, was associated with the C4/C1 ratio. Fifth, the sample comprised mixed curve types and directions, and kyphosis changes are more homogeneous across scoliosis subtypes, which may facilitate detection of TK associations while hindering detection of Cobb associations when curve types are combined. Distinguishing between these explanations will require longitudinal data. Consistent with our findings, a recent study of 50 female AIS patients also reported that Cobb angle showed no correlation with any postural control parameter [28].

A biomechanical basis for the TK–C4/C1 association is plausible. Thoracic kyphosis displaces the trunk’s centre of mass posteriorly, altering the geometry of postural control [9,10]. This posterior shift may increase the mechanical demands on lower-extremity proprioception and plantar cutaneous feedback, potentially upregulating somatosensory gain. By contrast, coronal plane curvature, even when substantial, may be compensated through counter-curvature and pelvic obliquity without fundamentally altering the anterior–posterior control strategy [10].

The present findings are not driven by a small subset of patients with abnormally elevated TK. The monotonic gradient across TK tertiles demonstrates that the C4/C1 ratio increases progressively from the Low TK group (mean TK within the hypokyphotic range typical of AIS) to the Mid TK group (mean TK within the lower end of the normative adolescent range) to the High TK group. Critically, the Mid TK group, whose thoracic kyphosis falls within the lower end of the normative range for adolescents (approximately 20°–40° [16,17]), already showed significantly higher C4/C1 ratios than the Low TK group (0.832 vs. 0.701, p = 0.040). This intermediate-tertile difference is pivotal: it demonstrates that the TK–C4/C1 association is not restricted to patients with pathological hyperkyphosis, but rather reflects a continuous relationship between sagittal alignment and performance under sensory perturbation that operates across the full spectrum of TK values observed in AIS, including the normokyphotic range. Thoracic kyphosis in AIS is best understood not as a categorical variable (hypokyphotic vs. normal vs. hyperkyphotic) but as a continuous morphometric parameter whose associations with postural control are graded rather than threshold-dependent.

Furthermore, the traditional view that AIS is invariably associated with thoracic hypokyphosis has been increasingly challenged by three-dimensional imaging studies. Using 3D reconstruction techniques, several groups have demonstrated that apparent “flat back” on 2D lateral radiographs frequently reflects vertebral axial rotation rather than true sagittal plane flattening [10,17]. Indeed, approximately 20–30% of AIS patients in large cohorts have TK values within or above the normal adolescent range [16,17]. The substantial TK variability observed in the present cohort (2°–53°, CV = 37.8%) is consistent with this emerging evidence and highlights the importance of evaluating sagittal alignment as a continuous morphometric parameter rather than applying a binary classification of “hypokyphotic” versus “normal.”

The Cobb–C5 correlation (ρ = 0.340, p = 0.028) merits comment. C5 (eyes closed, sway-referenced support surface) is the most challenging somatosensory+vestibular condition; Cobb angle was associated with better performance on C5 but not C4 (p = 0.073) or C6 (p = 0.850). This selective pattern may reflect the unique sensory demands of C5: when both vision and somatosensation are unreliable, vestibular function becomes critical. We speculate that patients with larger curves may develop enhanced vestibular reliance as a compensatory mechanism, although this hypothesis requires direct vestibular testing for confirmation. The evidence for vestibular involvement in AIS remains mixed, and further studies are needed [3,29].

### C4/C1 ratio and postural control: an indirect-only statistical pathway

Our finding that the C4/C1 ratio is associated with better postural control across multiple domains — stability, variability, and adaptation — is consistent with the sensory reweighting framework, in which the central nervous system dynamically adjusts the relative weighting of visual, vestibular, and somatosensory inputs according to their reliability [4]. Within this framework, individuals can adopt either an “inflexible” (persistent ankle reliance) or a “plastic” (proximal shift) proprioceptive strategy under challenging conditions [30]. Patients who maintain equilibrium effectively on the sway-referenced surface (high C4/C1) also exhibit better overall postural control, lower condition-to-condition variability, and a less precipitous decline across increasingly challenging SOT conditions.

Two interpretations of the C4/C1 ratio deserve consideration. Under the standard SOT convention [6,8], a higher C4/C1 ratio indicates relatively preserved performance when somatosensory input is disrupted by the sway-referenced surface. This may reflect effective use of remaining sensory information (visual and/or vestibular) to compensate for somatosensory perturbation. An alternative interpretation, which we explored, is that a high C4/C1 ratio may reflect a mathematical artifact: if baseline firm-surface performance (C1) is already low, the relative degradation on the sway-referenced surface (C4/C1) would appear smaller, paradoxically inflating the ratio. Our data argue against this alternative: C4/C1 was not correlated with C1 (ρ = −0.031, p = 0.844), and the C4/C1–MeanES association strengthened after partialling out C1 (partial Spearman ρ = 0.602). Furthermore, the C1 scores in our sample (mean 89.1 ± 5.9) are within the reported normative range for healthy adolescents [21,26], arguing against a generalized floor effect. Thus, the C4/C1 ratio in this cohort likely reflects between-condition sensory reweighting rather than a floor effect.

**Exploratory mediation analysis** showed a significant indirect statistical pathway (Sobel z = 2.49, p = 0.013) but a non-significant total association of TK with MeanES (ρ = 0.21, p = 0.18). The pattern of a significant indirect association (a × b) without a significant total association (c) is consistent with indirect-only mediation [27]. This can occur when the indirect and direct associations have opposing signs, resulting in a cancelled total association. In our data, the direct association (c’ = 0.03) was near zero after controlling for the C4/C1 ratio, while the indirect association via C4/C1 was positive and significant. This suggests that the C4/C1 ratio carries the positive association between TK and postural control, but the direct path (not mediated by C4/C1) is null or slightly negative. Several explanations are possible: low statistical power for the total association (N = 42), the multi-factorial nature of postural control, or the possibility that C4/C1 captures shared variance without representing a causal intermediate. As with all mediation analyses of cross-sectional data, the inferred pathways are statistical rather than causal, and temporal precedence cannot be established. We have also conducted a sensitivity analysis excluding C4 from the outcome variable to address mathematical coupling concerns (see the sensitivity analysis in section 3.6).

In AIS patients with lower TK (within the hypokyphotic range), worse performance on the sway-referenced platform (lower C4/C1) may reflect reduced capacity to use remaining sensory information when somatosensory input is disrupted. This interpretation is consistent with the clinical observation that the hypokyphotic effect of scoliosis reduces TK measurements, and therefore the direction of the association — lower TK associated with worse C4/C1 — is consistent with the expected direction of scoliosis-related functional impairment. The progressively flatter adaptation slope across TK tertiles (Low: −11.5; Mid: −8.8; High: −7.7) is consistent with greater resilience to sensory challenge in patients with TK values closer to the normative range (S1 Fig).

### Clinical implications — directions for future research

These findings suggest several directions for future investigation, though we emphasise that these are hypotheses to be tested in future studies rather than clinical recommendations.

First, the differential association pattern suggests that radiographic assessment of TK may provide complementary information to the traditional focus on Cobb angle in the functional evaluation of AIS. A patient with a modest coronal curve but substantial TK may have a fundamentally different postural control profile than one with a larger Cobb angle but preserved sagittal alignment. Future studies should examine whether combining TK and Cobb measurements improves the prediction of functional outcomes.

Second, the monotonic gradient across TK levels suggests that the relationship between TK and the C4/C1 ratio is continuous rather than categorical. Future work with larger cohorts could examine whether specific TK ranges are associated with clinically meaningful differences in postural control.

Third, the finding that the C4/C1 ratio is associated with better postural control suggests that interventions targeting the ability to maintain postural stability under somatosensory perturbation — such as proprioceptive training on unstable surfaces, vibration-based sensory enhancement, or textured insole interventions [27,30–34] — may be worthy of investigation in future intervention studies. Since somatosensory function is amenable to training [31,32], and scoliosis-specific exercises such as the Schroth method have shown positive effects on postural control and balance in AIS [33], future randomised controlled trials could test whether such interventions improve postural control in AIS patients identified by their sagittal profile. We emphasise that the present cross-sectional study cannot establish whether modifying these variables would confer benefit.

### Comparison with previous literature

Prior studies have reported altered sensory weighting in AIS compared with controls [25] and associations between somatosensory function and spinal asymmetry [29]. Recent work has demonstrated significant correlations between somatosensory evoked potentials and SOT-derived balance parameters in AIS [34], but did not examine sagittal parameter correlates. Haumont et al. [26] found that SOT performance was unrelated to Cobb angle, consistent with our null finding for Cobb–sensory ratio associations, but did not assess TK. To our knowledge, this is among the first studies to report a selective TK–C4/C1 association in AIS. The positive direction of this association — higher TK associated with relatively preserved sway-referenced platform performance — is consistent with the hypokyphotic effect of scoliosis: as scoliosis reduces TK, worse somatosensory-related performance is observed. The association remained significant after adjusting for Cobb angle (partial Spearman ρ from 0.416 to 0.475). This suppression pattern suggests that Cobb angle masks the TK–C4/C1 relationship in unadjusted analyses, likely because TK and Cobb are negatively correlated (ρ = −0.317, p = 0.041), consistent with the known thoracic scoliosis–hypokyphosis association [15,35].

### Limitations

Several limitations warrant consideration.

First, with N = 45 (n = 42 for primary analyses), the study is adequately powered for detecting moderate-to-large correlations (ρ ≥ 0.29 at 80% power) but limits subgroup analyses and may constrain the precision of tertile-based comparisons (n = 14 per group). The tertile comparisons are descriptive and should be interpreted with caution given the small group sizes and potential influence of individual observations.

Second, the absence of age-matched controls precludes normative comparisons, although published SOT norms are available [21,26].

Third, the cross-sectional design cannot establish causality; longitudinal studies are needed to determine whether TK progression is associated with changes in somatosensory-related performance over time.

Fourth, the C4/C1 ratio is a composite measure whose interpretation requires nuance: it reflects performance when somatosensory input is disrupted, and higher values may indicate effective use of remaining sensory information (visual and/or vestibular) rather than somatosensory function per se. This is partially addressed by corroboration with the somatosensory/visual preference ratio (C4/C2).

Fifth, the adaptation slope metric assumes approximately equal-interval difficulty steps across SOT conditions, an assumption that may not hold given the qualitatively different sensory perturbations introduced at each condition; this should be considered when interpreting slope differences.

Sixth, the mild-to-moderate cohort (median Cobb 15.3°, IQR 11.2°–20.0°) limits generalisability to severe surgical curves. Future studies should examine whether the TK–C4/C1 association is present in cohorts with more severe scoliosis.

Seventh, the behavioural somatosensory measure does not capture the underlying neural mechanism; future studies incorporating neurophysiological measures (e.g., somatosensory evoked potentials, EMG-based assessment of proprioceptive processing) are warranted.

Eighth, radiographic measurements were performed by a single rater; although intra-rater reliability was excellent (ICC > 0.95), inter-rater reliability was not assessed.

Ninth, full-spine magnetic resonance imaging (MRI) was not performed; occult neural axis abnormalities (e.g., Chiari malformation, syringomyelia, tethered cord) cannot be definitively excluded. However, all participants were neurologically intact on clinical examination, had no cutaneous stigmata of spinal dysraphism, and met SRS diagnostic criteria for AIS. The absence of MRI is a limitation common to many clinical AIS studies, and the inclusion criteria used (idiopathic curve pattern, normal neurological examination) carry a low pretest probability of neural axis abnormalities in this cohort.

Tenth, curve type (single thoracic, thoracolumbar/lumbar, double major) was not included as a covariate in the primary analyses due to limited subgroup sizes; future studies should examine whether the TK–C4/C1 association differs by curve type.

Eleventh, TK was measured on conventional 2D lateral radiographs rather than in the true sagittal plane of each vertebra, which may be affected by axial rotation; 3D reconstruction techniques could provide more accurate sagittal alignment measurements.

Twelfth, eight patients (17.8%) were aged 8–9 years, falling within the juvenile idiopathic scoliosis (JIS) range by strict SRS classification. Although sensitivity analyses confirmed that the primary findings were robust to their exclusion (see the Robustness checks section and S3 Table in S1 File), the inclusion of JIS patients may limit the generalizability of findings to strictly defined AIS populations. This limitation is mitigated by evidence that JIS and AIS patients show comparable outcomes when managed under the same protocol [20], and by the 2024 Chinese AIS guideline recommendation for screening at ages 8–18 [19].

Thirteenth, all participants were assessed at the time of initial screening, before initiating any scoliosis-specific intervention. While this eliminates treatment history as a confound, it also means that the findings reflect the pre-treatment state and may not generalise to patients undergoing active treatment.

Fourteenth, the SOT measures only anteroposterior (sagittal plane) sway and does not capture mediolateral sway, which may be relevant given the coronal plane asymmetry in AIS.

Fifteenth, the number of statistical tests performed across primary and exploratory analyses, while controlled within families via FDR correction, may inflate the overall familywise error rate. The exploratory analyses should be interpreted with appropriate caution.

## Conclusions

In a cohort of 42 patients with adolescent idiopathic scoliosis, thoracic kyphosis was independently associated with the C4/C1 ratio, which in turn was associated with better postural control across multiple domains. By contrast, coronal Cobb angle showed no association with sensory integration but was positively associated with firm-surface equilibrium scores. This differential pattern — TK associated with performance under sensory perturbation, Cobb associated with firm-surface stability — indicates that sagittal and coronal spinal parameters are associated with postural control through distinct pathways. The TK–C4/C1 association was robust to adjustment for multiple confounders and exhibited a monotonic gradient across TK tertiles. These findings identify sagittal plane morphology as a correlate of sensory-motor function in AIS and suggest that future research should examine whether interventions targeting performance under somatosensory perturbation may be particularly relevant for patients identified by their sagittal profile.

## Supporting information

S1 File S1–S4. Supplementary Tables, Data, and Code. This ZIP archive contains: S1 Table (Partial Spearman Correlations: TK → C4/C1 and TK → C4/C2), S2 Table (Spearman Correlations: Spinal Parameters vs. SOT Condition Scores), S3 Data (De-identified Participant-Level Dataset with 45 enrolled participants), S3 Data Codebook (Data Dictionary with variable definitions and coding schemes), S4 Table (Sensitivity Analysis: Exclusion of Patients Aged <10 Years, N = 34), and S4 Code (Python 3.12 Analysis Scripts).
(ZIP)

S1 Fig Equilibrium Score Profiles by TK Tertile (mean ± SEM). C1–C6 equilibrium score profiles for Low TK (≤22°), Mid TK (22°–34°), and High TK (≥34°) groups, presented as mean ± SEM. Note the progressively flatter profile with increasing TK, reflecting relatively preserved performance across sensory challenges.
(PNG)
