# Supplementary material for: Thoracic kyphosis is associated with somatosensory dependence and postural control in adolescent idiopathic scoliosis
Source: PLoS One. 2026 Oct 23;21(10):e0361226. doi: 10.1371/journal.pone.0361226 (PMC13693936; doi:10.1371/journal.pone.0361226)
Supplement: Supplementary file 1 [file pone.0361226.s004.docx]

# Response to Reviewers

**Manuscript:** PONE-D-26-34577 — "Thoracic Kyphosis Is Associated with Somatosensory Dependence and Postural Control in Adolescent Idiopathic Scoliosis"

**Journal:** *PLOS ONE*

**Date:** September 17, 2026

We sincerely thank the Academic Editor and both reviewers for their thorough and constructive evaluation. We have addressed every point raised. Below we provide a point-by-point response; reviewer comments are in **bold**, our responses follow. All changes to the manuscript are described in detail.

## Part A: Response to Journal Requirements

### J1. PLOS ONE style requirements

**Response:** We have reviewed the PLOS ONE formatting sample documents and updated the manuscript accordingly. The manuscript now follows PLOS ONE style, including: sentence case headings, "Fig" (not "Figure") for figure citations, Vancouver reference style, IMRaD structure, and structured abstract.

### J2–J3. Data Availability — public access

**Response:** We have revised the Data Availability statement. The anonymized dataset and analysis code are provided as Supporting Information files (S3 Data and S4 Code). The data are made publicly available under a Creative Commons Attribution 4.0 International License (CC BY 4.0) with no access restrictions. The statement now reads:

*"The anonymized participant-level dataset and analysis code underlying the findings of this study are provided as Supporting Information files (S3 Data and S4 Code). The dataset has been de-identified by removing all direct identifiers (name, medical record number, and date of birth). Individual-level data points for all variables reported in Tables 1–4 and Supplementary Tables S1–S2 are included. The data are publicly available under a Creative Commons Attribution 4.0 International License (CC BY 4.0) with no access restrictions."*

### J4. Author affiliations

**Response:** Wenjun Jiang is affiliated with Suzhou Vocational Health College, Suzhou, China. All other five authors (Ningnan Zhu, Peng Zhang, Jiahuan Wu, Sheng Wang, Xueliang Wu) are affiliated with the same institution (Suzhou Research Center of Medical School, Suzhou Hospital, Affiliated Hospital of Medical School, Nanjing University, Suzhou, China). Each author is linked to their respective affiliation in the author list. This is confirmed.

### J5. Supplementary tables S1 and S2 removed from manuscript file

**Response:** We have removed the in-text content of S1 Table and S2 Table from the manuscript body. These tables are now provided as separate Supporting Information files. Each has a legend listed in the Supporting Information section after the references.

### J6. Reviewer citation recommendations

**Response:** We have carefully evaluated all references cited by the reviewers. Where relevant, we have incorporated them (see specific responses below). The reviewers did not recommend specific additional citations beyond those already in the manuscript.

## Part B: Response to Reviewer #1

### R1.1. Keywords are not MESH terms

**Response:** We have verified that our keywords map to the following MeSH terms: Scoliosis (MeSH D012600), Kyphosis (MeSH D007738), Postural Balance (MeSH D004856), Proprioception (MeSH D011434), Feedback, Sensory (MeSH D005246), Somatosensory Disorders (MeSH D020886). The Sensory Organization Test is a specific clinical protocol and does not have a direct MeSH term. We have retained the current keyword list as it is consistent with MeSH terminology.

### R1.2. Avoid "deformity" — use "structural misalignment"

**Response:** We have replaced "coronal deformity" with "coronal structural misalignment" throughout the manuscript. The term "deformity" is retained only in the journal name "Spine Deformity" in the reference list, which is a proper noun.

### R1.3–5. Abstract improvements

**Response:** We have revised the Abstract to: (a) specify that the association was examined in AIS patients; (b) state that there was no selection criterion related to kyphosis; (c) specify the recruitment setting (outpatient rehabilitation clinic of a university-affiliated hospital); (d) define all abbreviations on first use (ES, FDR, C1, C4); and (e) explain what the C4/C1 ratio represents.

### R1.6. Specify partial correlations are non-parametric (partial Spearman)

**Response:** We have clarified throughout the manuscript that partial correlations are partial Spearman correlations. The term "partial Spearman" is now used consistently.

### R1.7–8. Define abbreviations and explain C4/C1 in Abstract

**Response:** All abbreviations are now defined on first use in the Abstract. The C4/C1 ratio is explicitly explained: "The C4/C1 ratio reflects the ability to maintain postural stability when somatosensory input is disrupted by a sway-referenced support surface (C4) relative to a fixed surface (C1)."

### R1.9. "Complementary dissociation pattern" → rephrase

**Response:** We have replaced "dissociation" with "differential association pattern" throughout the manuscript. The Abstract Interpretation now reads: "TK and Cobb angle showed differential associations with postural control."

### R1.10. Avoid causal implications in Abstract

**Response:** We have revised the Abstract Interpretation to remove causal language. The phrase "with implications for phenotype-specific rehabilitation" has been replaced with "which may inform future research on phenotype-specific assessment strategies."

### R1.11. Explain SOT six conditions in Introduction

**Response:** The six SOT conditions are already described in paragraph 3 of the Introduction with full descriptions of C1–C6. This was already present in the manuscript.

### R1.12. "driven primarily by coronal deformity" → avoid causal language

**Response:** Changed to "associated primarily with coronal curvature (Cobb angle), with sagittal alignment (TK), or with both."

### R1.13. "(thoracic hypokyphosis accompanies curve progression)" → add citation

**Response:** We have added citations [15,16,17] to support this statement. References 15 (Negrini et al., 2018 SOSORT guidelines), 16 (Mac-Thiong et al., 2003), and 17 (Yong et al., 2012) all document the association between thoracic hypokyphosis and curve progression in AIS.

### R1.14. Explain C4/C1 ratio and justify interest in Introduction

**Response:** The C4/C1 ratio is already explained in paragraph 4 of the Introduction, including its physiological interpretation and justification. This was already present in the manuscript.

### R1.14a. Explain how sensorimotor integration was tested

**Response:** We have revised the sentence to explicitly state how sensorimotor integration was assessed: "Recent work has highlighted sensorimotor integration deficits as a potential aetiological factor in AIS, assessed via diffusion tensor imaging, which has revealed disruptions in both somatosensory afferent and motor efferent pathways in AIS [7]." The method of assessment is now stated within the same sentence as suggested.

### R1.15. Results summary in Introduction → move to Discussion

**Response:** We have reviewed the Introduction and confirmed that no results summary is presented before the Methods or Results sections. The Introduction describes the study aims and hypotheses without previewing results.

### R1.16. Age 8 — technically juvenile (JIS)

**Response:** We acknowledge this concern. Eight patients (17.8%) were aged 8–9 years. We have retained them in the primary analysis with explicit justification (Section 2.1): the 2024 Chinese AIS guideline recommends screening at ages 8–18 [19]; a prior study showed no significant difference in outcomes between JIS and AIS patients treated after age 10 [20]; and sensitivity analyses excluding these patients confirmed robustness (the Robustness checks section (3.7), S4 Table). This limitation is acknowledged in the Limitations section.

### R1.17. Consecutive recruitment

**Response:** We have clarified in Section 2.1 that patients were "prospectively and consecutively recruited" from the specified date range.

### R1.18. Exclusion diagnoses — self-reported or standard screen?

**Response:** We have clarified: "Exclusion criteria were assessed through clinical examination and medical history review by a rehabilitation physician."

### R1.19. Report curve angles and curve types

**Response:** Curve angles and curve types are reported in Table 1 and in Section 3.2 (Participant characteristics). The cohort had a median Cobb angle of 15.3° (IQR 11.2°–20.0°) and curve types were: single thoracic 21.4%, thoracolumbar/lumbar 73.8%, double major 4.8%.

### R1.19a. Which AVT was measured

**Response:** Table 1 now specifies "AVT (major curve, mm)." The apical vertebral translation was measured for the major curve only. We have clarified this in the table header.

### R1.20. C7-CSVL — sagittal or frontal?

**Response:** We have clarified in Section 2.2: "C7–central sacral vertical line (C7-CSVL, measured in the frontal (coronal) plane)." This is also noted in Table 1.

### R1.21. Risser — American or European?

**Response:** We have specified: "Skeletal maturity was assessed using the Risse plus (Risser+) classification, coded as an ordinal scale: 0− = 1, 0+ = 2, 1 = 3, 2 = 4, 3 = 5, 3/4 = 6, 4 = 7, 5 = 8."

### R1.22. Standardized imaging position

**Response:** We have added: "Radiographs were acquired with participants standing in a relaxed, upright position with arms folded across the chest at approximately 45° to the horizontal plane, feet shoulder-width apart, and gaze directed forward."

### R1.23. SOT condition testing order

**Response:** We have specified: "The six conditions were administered in the standard fixed order (C1 through C6), with each condition performed in triplicate."

### R1.24. Why θ_max only in sagittal plane?

**Response:** We have added: "The SOT equilibrium score measures sway in the anteroposterior (sagittal) plane, as this is the primary plane of postural control during quiet stance [6,21]." This is also acknowledged as a limitation (Limitation 14): "the SOT measures only anteroposterior sway and does not capture mediolateral sway."

### R1.25. Announced 5 ratios but defined 4

**Response:** We have corrected this. The Methods section now consistently lists five sensory ratios: visual dependence (C2/C1), somatosensory dependence (C4/C1), vestibular dependence (C5/C1), visual preference (C3/C1), and somatosensory/visual preference (C4/C2). All five are reported in Table 2.

### R1.26. PostRes formula — insufficient detail

**Response:** We have expanded the PostRes definition: "Computed as the residual variance from a participant-level ordinary least squares regression of the six condition-level equilibrium scores on condition number (ES ~ condition_number). Higher values indicate greater deviation from the expected linear decline across conditions, representing a larger postural control deficit."

### R1.27. CV — individual trials or mean of three?

**Response:** We have clarified: "Calculated using the mean of the three trials per condition."

### R1.27a. Table 2 — inclusion of derived postural control indices

**Response:** Table 2 now includes all derived postural control indices (PostRes, CV, and Slope) alongside the condition-level equilibrium scores and sensory dependence ratios. StratShf has been removed (see R1.28).

### R1.28. Strategy Shift — how scored?

**Response:** We appreciate this request for clarification. Upon verification, we found that StratShf could not be fully traced to the per-condition strategy scores from the original NeuroCom device data, and this index did not enter any primary or secondary outcome analysis. To ensure complete reproducibility, we have removed StratShf from the manuscript (Methods 2.4, Table 2, Table 4). The FDR correction family for tertile comparisons was correspondingly adjusted from 8 to 7 group comparisons, with no change to the significance conclusions. All other results are unaffected.

### R1.29. SOT measurement properties — references

**Response:** We have added: "References for the measurement properties of the SOT, including test-retest reliability of the composite score and sensory ratios, are provided in the published literature [6,21,8]."

### R1.30. SOT testing relative to radiographs

**Response:** We have clarified: "SOT testing and radiography were performed on the same day, with the SOT administered first to avoid fatigue effects from prolonged standing during radiographic positioning."

### R1.31. Avoid "SomDep" abbreviation

**Response:** We have removed the "SomDep" abbreviation. The full term "somatosensory dependence ratio" or "C4/C1 ratio" is used consistently throughout.

### R1.32. Mean ES vs Mean EQ

**Response:** We have standardized all references to "MeanES" (Mean Equilibrium Score) throughout the manuscript. This variable is defined in Section 2.4 with coding direction (higher = better).

### R1.33–34. Mediation framework figure and missing data

**Response:** A mediation framework figure (Fig 3) is provided. A "Missing data" subsection (Section 3.1) has been added to the beginning of Results.

### R1.35. Report n = 41/42 rather than N = 45

**Response:** The Abstract now reports both the enrolled sample (N = 45) and the analytic sample (n = 42 for primary analyses). Sample sizes are explicitly noted for each analysis throughout.

### R1.36. Mild scoliosis — generalizability

**Response:** This is acknowledged as Limitation 6: "the mild-to-moderate cohort (median Cobb 15.3°, IQR 11.2°–20.0°) limits generalisability to severe surgical curves."

### R1.37–40. Table format — medians and IQR

**Response:** Tables 1 and 2 already report both mean ± SD and median (IQR). We have retained this dual reporting as it provides complementary information for readers.

### R1.41. Report only one partial correlation model

**Response:** We have streamlined the partial correlation reporting. The primary model (controlling for Cobb, age, and BMI jointly) is reported as the main result. Bivariate correlations are reported for comparison. The multiple single-covariate models have been removed from the main text.

### R1.42. Avoid "dissociation"

**Response:** Replaced with "differential association pattern" or "differential pattern" throughout.

### R1.43. Avoid "raw" for condition scores

**Response:** Replaced "raw SOT scores" with "SOT condition scores" or "condition-level equilibrium scores."

### R1.43a. Minor text edits

**Response:** We have deleted the phrase "a finding examined further in the Discussion" from the Results section. Text describing the complementary pattern of Cobb–TK associations has been moved to the Discussion where it can be discussed with appropriate context.

### R1.44–45. Tertile analysis — not a threshold; Kruskal-Wallis not a trend test

**Response:** We have revised the language throughout: (a) removed references to "threshold" and replaced with "tertile boundary"; (b) removed "non-significant trend" language; (c) added explicit caveat: "Tertile boundaries are data-derived and should not be interpreted as clinically meaningful thresholds"; (d) "monotonic gradient" is used instead of "dose-response."

### R1.45a. Reviewer text suggestion for tertile results

**Response:** We have adopted the reviewer's suggested clearer wording for the monotonic gradient description in Section 3.4: "With increasing TK, the adaptation slope became progressively less negative from Low TK to High TK, and response variability decreased from Low to High."

### R1.46. Report Cobb→C1 partial correlation

**Response:** These results are presented in the Discussion (Section 4.1); the Cobb–C1 association remained significant after controlling for age and Risser stage: "The Cobb–C1 association remained significant after controlling for age and Risser stage (partial Spearman ρ = 0.41, p = 0.008)."

### R1.47. ES measures sagittal plane sway → may be more sensitive to TK

**Response:** This has been added as the fourth explanation for the Cobb–firm-surface finding in Section 4.1: "Fourth, the SOT equilibrium score measures anteroposterior (sagittal plane) sway, which may be more sensitive to sagittal plane changes (TK) than to coronal plane changes (Cobb angle)."

### R1.48. Mixed curve types may hinder Cobb detection

**Response:** This has been added as the fifth explanation in Section 4.1: "Fifth, the sample comprised mixed curve types and directions, and kyphosis changes are more homogeneous across scoliosis subtypes, which may facilitate detection of TK associations while hindering detection of Cobb associations when curve types are combined."

### R1.48a. Curve type as a limitation

**Response:** This limitation has been added (Limitation 10): "Tenth, curve type (single thoracic, thoracolumbar/lumbar, double major) was not included as a covariate in the primary analyses due to limited subgroup sizes; future studies should examine whether the TK–C4/C1 association differs by curve type."

### R1.49. 22°–34° as normal range — cite source

**Response:** We have cited references [16] (Mac-Thiong et al., 2003) and [17] (Yong et al., 2012) for the normative adolescent TK range. We have also clarified that the range described in the revised manuscript (approximately 20°–40°) represents the lower end of the normative adolescent TK range [16,17].

### R1.50. "consequence" → avoid causal language

**Response:** Changed to "biomechanical associations with postural control."

### R1.51. 2D TK measurement affected by axial rotation — limitation

**Response:** This is acknowledged as Limitation 11: "TK was measured on conventional 2D lateral radiographs rather than in the true sagittal plane of each vertebra, which may be affected by axial rotation; 3D reconstruction techniques could provide more accurate sagittal alignment measurements."

### R1.52. Vestibular function and Cobb angle

**Response:** We have added a discussion of the Cobb–C5 correlation in Section 4.1, speculating that "patients with larger curves may develop enhanced vestibular reliance as a compensatory mechanism." We acknowledge that "the evidence for vestibular involvement in AIS remains mixed, and further studies are needed [3,29]."

### R1.53. High C4/C1 — compare C1 to adolescent norms

**Response:** We have added: "Furthermore, the C1 scores in our sample (mean 89.1 ± 5.9) are within the reported normative range for healthy adolescents [21,26], arguing against a generalized floor effect."

### R1.54. Complete mediation figure

**Response:** Fig 3 now presents the complete mediation analysis framework with all paths (a, b, c, c') and coefficients, including the Sobel test result.

### R1.55. Indirect effect — suppression or mediation-only?

**Response:** We have clarified in the Discussion: "The pattern of a significant indirect association (a × b) without a significant total association (c) is consistent with indirect-only mediation [27]. This can occur when the indirect and direct associations have opposing signs, resulting in a cancelled total association. In our data, the direct association (c' = 0.03) was near zero after controlling for the C4/C1 ratio."

### R1.56. "Higher TK = adapted" — wording

**Response:** We have revised this section. The text now reads: "In AIS patients with lower TK (within the hypokyphotic range), worse performance on the sway-referenced platform (lower C4/C1) may reflect reduced capacity to use remaining sensory information when somatosensory input is disrupted. This interpretation is consistent with the clinical observation that the hypokyphotic effect of scoliosis reduces TK measurements, and therefore the direction of the association — lower TK associated with worse C4/C1 — is consistent with the expected direction of scoliosis-related functional impairment."

### R1.57. Avoid "threshold" discussion

**Response:** Removed all threshold-related language. Replaced with "tertile boundary" and added explicit caveats. The discussion now frames TK as a continuous morphometric parameter.

### R1.58. Third clinical implication → future research direction

**Response:** The Clinical Implications section is now titled "Clinical implications — directions for future research" and all statements are framed as hypotheses for future investigation. We explicitly state: "We emphasise that the present cross-sectional study cannot establish whether modifying these variables would confer benefit."

### R1.59. "determinant" in conclusion

**Response:** Changed to "correlate": "These findings identify sagittal plane morphology as a correlate of sensory-motor function in AIS."

### R1.60. Fig 1 legend — spell out OLS

**Response:** The legend now reads: "ordinary least squares (OLS) linear fit."

### R1.61. Fig 2 — individual observations influence

**Response:** The Fig 2 legend already includes: "Note that the tertile comparisons are descriptive and may be influenced by individual observations given the small group sizes."

### R1.61a. Fig 1 and threshold interpretation

**Response:** We agree that Fig 1 does not support a threshold interpretation of the TK–C4/C1 relationship. All threshold-related language has been removed from the manuscript (see R1.44–45 and R1.57). The association is discussed as a continuous gradient.

### R1.62. S1 Fig — non-linear, remove regression slopes

**Response:** The S1 Fig legend has been revised: "The profiles are presented descriptively. Linear regression slopes are not reported because the condition-to-condition difficulty steps are not necessarily equal-interval."

### R1.62a. Discussion section length and structure

**Response:** The Discussion section has been restructured and subsection headings reorganised for clarity. The first sentence of each subsection has been reviewed and shortened where appropriate.

## Part C: Response to Reviewer #2

### Major Comments

### R2.M1. C4/C1 interpretation — inconsistent with standard SOT

**Response:** We have substantially revised the C4/C1 interpretation throughout the manuscript. Key changes:

1. In Section 2.4, we added a "Note on SOT sensory ratio nomenclature" explaining that our functional labelling (naming ratios by the modality being challenged) differs from the manufacturer's convention (SOM = C2/C1, VIS = C4/C1), and that all ratios were computed directly from condition-level ES values.

2. We clarified that the C4/C1 ratio "reflects the ability to maintain postural stability when proprioceptive and plantar cutaneous feedback are rendered unreliable while visual and vestibular information remain available. A higher C4/C1 ratio does not necessarily reflect better somatosensory utilisation per se; it may reflect effective compensatory reliance on visual and/or vestibular inputs."

3. A direction note was added at the beginning of Section 3.3: "A higher C4/C1 ratio indicates relatively preserved performance on the sway-referenced platform, i.e., less degradation when somatosensory input is disrupted."

4. Alternative interpretations are discussed in the Discussion (Section 4.2).

### R2.M2. Mediation analysis — mathematical coupling

**Response:** We agree that mathematical coupling is a concern because C4 contributes to both the mediator (C4/C1) and the outcome (MeanES). We have addressed this in two ways:

1. **Sensitivity analysis** (Section 3.7): We repeated the mediation analysis using an outcome that excludes C4 — the mean of C1, C2, C3, C5, and C6 (MeanES_noC4). The indirect pathway was attenuated (Sobel z = 1.83, p = 0.067), consistent with the expected reduction in statistical power when C4 is removed from the outcome. The direction of the indirect association remained positive, suggesting that the mediation finding is not solely attributable to mathematical coupling, though the attenuation to marginal significance indicates that shared C4 variance contributes to the strength of the primary mediation result. We acknowledge that a partial contribution of mathematical coupling cannot be fully excluded given p = 0.067.

2. We have also demonstrated that partialling out C1 from the C4/C1–MeanES correlation **strengthens** the association (partial Spearman ρ = 0.602), confirming that the correlation is not a mathematical artifact of shared C1 variance.

3. The mediation analysis is explicitly labeled as "exploratory" throughout.

### R2.M3. Cross-sectional mediation — avoid causal language

**Response:** We have revised the mediation language throughout:

1. The mediation analysis is labeled "Exploratory mediation analysis" in all sections.

2. We emphasize: "As with all mediation analyses of cross-sectional data, the inferred pathways are statistical rather than causal, and temporal precedence cannot be established."

3. The term "indirect-only statistical pathway" is used instead of "mediation pathway."

4. The Abstract now states: "The C4/C1 ratio statistically carried the association between TK and postural control via an indirect-only pathway."

### R2.M4. Sample size and multiplicity — distinguish primary vs. exploratory

**Response:** We have clearly distinguished between:

1. **Primary (prespecified) analyses** (Section 2.5): Three analyses specified a priori — (1) Spearman correlation between TK and C4/C1; (2) partial Spearman correlation controlling for Cobb, age, and BMI; (3) Kruskal-Wallis test across TK tertiles.

2. **Secondary/exploratory analyses** (Section 2.5): All other analyses, including the mediation analysis, are explicitly labeled as exploratory.

3. **Multiple testing correction families** are defined: (a) five sensory ratios (5 tests); (b) six SOT condition scores (6 tests); (c) seven tertile group comparisons (7 tests); (d) four postural control indices (4 tests). FDR correction is applied within each family.

### R2.M5. Tertile-derived threshold — avoid

**Response:** We have removed all references to a "threshold" of 22°. The tertile boundaries are now described as "data-derived and should not be interpreted as clinically meaningful thresholds." The Discussion frame associated TK as a continuous morphometric parameter with graded associations.

### R2.M6. Confounding by treatment and maturation

**Response:** We have clarified:

1. All participants were "newly screened and had not yet initiated scoliosis-specific exercise, physiotherapy, or brace treatment at the time of assessment" (Section 2.1).

2. "As all participants were pre-treatment, no braces were worn during SOT testing" (Section 2.3).

3. Covariate rationale is stated: "Covariates were selected for their known or plausible influence on postural control: Cobb angle (coronal curvature severity), age (developmental maturation), and BMI (body habitus effects on postural control)."

4. A partial Spearman correlation controlling for age and Risser stage was performed for the Cobb–C1 association (Discussion Section 4.1).

### R2.M7. Derived postural indices — derivation details

**Response:** We have expanded the derivation of all postural control indices:

1. **PostRes:** "Computed as the residual variance from a participant-level ordinary least squares regression of the six condition-level equilibrium scores on condition number (ES ~ condition_number)."

2. **Slope:** "The regression coefficient from the participant-level linear regression of ES on condition number (C1 → C6). Note: this metric assumes approximately equal-interval difficulty steps across SOT conditions (see Limitations)."

3. **Strategy Shift:** After verification, we were unable to fully trace StratShf to the per-condition strategy scores from the original NeuroCom device data. As this index did not enter any primary or secondary outcome analysis, we have removed it from the manuscript to ensure complete reproducibility.

4. **CV:** "Calculated using the mean of the three trials per condition."

### R2.M8. Clinical implications — reframe as hypotheses

**Response:** The Clinical Implications section has been retitled "Clinical implications — directions for future research." All statements are framed as hypotheses: "These findings suggest several directions for future investigation, though we emphasise that these are hypotheses to be tested in future studies rather than clinical recommendations." We explicitly state: "We emphasise that the present cross-sectional study cannot establish whether modifying these variables would confer benefit."

### Minor Comments

### R2.m1. "predicts" → "is associated with"

**Response:** Replaced throughout the manuscript. "Predicts" is retained only in the statistical sense (e.g., "regression model predicted") where appropriate.

### R2.m2. "dose-response" → "monotonic gradient"

**Response:** Replaced throughout. The term "monotonic gradient across TK tertiles" is now used consistently.

### R2.m3. Don't call 22° a clinical threshold

**Response:** Removed. The boundary is described as "the 33rd percentile of the sample distribution" and "should not be interpreted as a clinically meaningful threshold."

### R2.m4. "genuine sensory reweighting" → soften

**Response:** Changed to "likely reflects between-condition sensory reweighting."

### R2.m5. "first demonstration" → more cautious

**Response:** Changed to "among the first studies to report a selective TK–C4/C1 association in AIS."

### R2.m6. Cite related study from same cohort

**Response:** We have reviewed the manuscript for any references to unpublished work from the same cohort. The manuscript does not contain references to related studies from this cohort. If there is a related publication, we will add the citation once the details are available.

### R2.m7. Distinguish primary vs. secondary outcomes

**Response:** The Methods section (2.5) now clearly distinguishes "Primary (prespecified) analyses" from "Secondary/exploratory analyses."

### R2.m8. Rationale for each covariate

**Response:** Added: "Covariates were selected for their known or plausible influence on postural control: Cobb angle (coronal curvature severity), age (developmental maturation), and BMI (body habitus effects on postural control)."

### R2.m9. Missing data description

**Response:** A "Missing data" subsection (Section 3.1) has been added: "Of the 45 enrolled patients, 42 had complete radiographic and SOT data for all primary analyses. Three patients had incomplete SOT data due to equipment malfunction during one testing session (one patient missing C5 and C6 trials; two patients missing C6 trials only). One additional subject was excluded from partial correlation models due to missing BMI (weight measurement inadvertently omitted). Missingness was not significantly associated with any demographic or clinical variable (age, sex, Cobb angle, TK; all p > 0.30)."

### R2.m10. Provide anonymized data and code

**Response:** The anonymized participant-level dataset (S3 Data) and analysis code (S4 Code) are provided as Supporting Information files. The data dictionary (S3 Data Codebook) is also included.

We believe these revisions have substantially strengthened the manuscript and addressed all concerns raised by the editor and both reviewers. We are grateful for the thorough and constructive review process.

Respectfully,

Wenjun Jiang^1*^, Ningnan Zhu^2*^, Peng Zhang^2^, Jiahuan Wu^2^, Sheng Wang^2†^, Xueliang Wu^2†^

^1^ Suzhou Vocational Health College, Suzhou, China

^2^ Suzhou Research Center of Medical School, Suzhou Hospital, Affiliated Hospital of Medical School, Nanjing University, Suzhou, China

\* Co-first authors; † Co-corresponding authors
