# Supplementary material for: Thoracic kyphosis is associated with somatosensory dependence and postural control in adolescent idiopathic scoliosis
Source: PLoS One. 2026 Oct 23;21(10):e0361226. doi: 10.1371/journal.pone.0361226 (PMC13693936; doi:10.1371/journal.pone.0361226)
Supplement: Supplementary file 2 [file pone.0361226.s001.zip › S1_Table.docx]

# S1 Table. Partial Spearman Correlations: TK → C4/C1 and TK → C4/C2, Controlling for Confounders

| Model | Partial ρ | p | Notes |
| --- | --- | --- | --- |
| TK → C4/C1 (bivariate) | 0.416 | 0.006 | n = 42 |
| TK → C4/C1 (+ Cobb, Age, BMI) | 0.480 | 0.002 | n = 41; primary model |
| TK → C4/C2 (bivariate) | 0.428 | 0.005 | n = 42 |
| TK → C4/C2 (+ Cobb, Age, BMI) | 0.466 | 0.002 | n = 41 |

*Note: n = 41 for all partial correlation models (one subject excluded due to missing BMI). The primary model controls for Cobb angle, age, and BMI jointly. The partial correlation remains significant after covariate adjustment, indicating that the TK–C4/C1 association is not confounded by coronal deformity, developmental maturation, or body habitus. Both C4/C1 and C4/C2 are reported for completeness. FALL trials were scored as 0 per standard SOT convention.*
