# Supplementary material for: Thoracic kyphosis is associated with somatosensory dependence and postural control in adolescent idiopathic scoliosis
Source: PLoS One. 2026 Oct 23;21(10):e0361226. doi: 10.1371/journal.pone.0361226 (PMC13693936; doi:10.1371/journal.pone.0361226)
Supplement: Supplementary file 2 [file pone.0361226.s001.zip › S2_Table.docx]

# S2 Table. Spearman Correlations: Spinal Parameters vs. SOT Condition Scores

| SOT Condition | TK ρ | TK p | Cobb ρ | Cobb p |
| --- | --- | --- | --- | --- |
| C1 (Eyes open, fixed support, fixed visual surround) | −0.160 | 0.312 | **0.543** | **<0.001** |
| C2 (Eyes closed, fixed support) | −0.213 | 0.176 | **0.606** | **<0.001** |
| C3 (Eyes open, fixed support, sway-referenced visual surround) | −0.155 | 0.327 | **0.545** | **<0.001** |
| C4 (Eyes open, sway-referenced support, fixed visual surround) | 0.292 | 0.061 | 0.280 | 0.073 |
| C5 (Eyes closed, sway-referenced support) | 0.096 | 0.547 | **0.340** | **0.028** |
| C6 (Eyes open, sway-referenced support, sway-referenced visual surround) | **0.332** | **0.032** | 0.030 | 0.850 |

*Values are Spearman ρ; n = 42. Bold = p < 0.05. Cobb angle is associated with firm-surface performance (C1–C3); TK is associated with the most challenging condition (C6). Note the complementary pattern of associations. FALL trials were scored as 0 per standard SOT convention.*
