# Supplementary material for: Thoracic kyphosis is associated with somatosensory dependence and postural control in adolescent idiopathic scoliosis
Source: PLoS One. 2026 Oct 23;21(10):e0361226. doi: 10.1371/journal.pone.0361226 (PMC13693936; doi:10.1371/journal.pone.0361226)
Supplement: Supplementary file 2 [file pone.0361226.s001.zip › S3_Data_Codebook.docx]

# S3 Data. De-identified Participant-Level Data

**Data Dictionary (Codebook)**

| Column | Description | Type | Values |
| --- | --- | --- | --- |
| Participant_ID | De-identified participant code | String | 1–45 |
| Age_years | Age range (binned to protect privacy) | String | 8–10, 11–13, 14–16, 17–18 |
| Sex_F1_M0 | Biological sex | Integer | 1 = Female, 0 = Male |
| Height_cm | Standing height | Float | cm |
| Weight_kg | Body weight | Float | kg |
| BMI | Body mass index | Float | kg/m² |
| Risser_US_0to5 | Skeletal maturity (Risser plus (Risser+), ordinal) | Integer | 0 = not recorded; 1 = 0−; 2 = 0+; 3 = 1; 4 = 2; 5 = 3; 6 = 3/4; 7 = 4; 8 = 5 |
| Cobb_angle_deg | Major Cobb angle | Float | degrees |
| TK_T5T12_deg | Thoracic kyphosis (T5–T12) | Float | degrees |
| LL_L1S1_deg | Lumbar lordosis (L1–S1) | Float | degrees |
| C7CSVL_frontal_mm | C7–central sacral vertical line (frontal plane) | Float | mm |
| AVT_major_curve_mm | Apical vertebral translation of major curve | Float | mm |
| Curve_direction_L1_R0 | Curve convexity direction | Integer | 1 = Left-convex, 0 = Right-convex |
| Curve_type | Curve pattern | Integer | 1 = Single thoracic, 2 = Thoracolumbar/lumbar, 3 = Double major |
| C1_ES | SOT Condition 1 equilibrium score | Float | 0–100 |
| C2_ES | SOT Condition 2 equilibrium score | Float | 0–100 |
| C3_ES | SOT Condition 3 equilibrium score | Float | 0–100 |
| C4_ES | SOT Condition 4 equilibrium score | Float | 0–100 |
| C5_ES | SOT Condition 5 equilibrium score | Float | 0–100 |
| C6_ES | SOT Condition 6 equilibrium score | Float | 0–100 |
| C2C1 | Visual dependence ratio | Float | — |
| C4C1 | Somatosensory dependence ratio | Float | — |
| C5C1 | Vestibular dependence ratio | Float | — |
| C4C2 | Somatosensory/visual preference ratio | Float | — |
| C3C1 | Visual preference ratio | Float | — |
| MeanES | Mean equilibrium score (C1–C6) | Float | 0–100 |
| PostRes | Postural reserve deficit | Float | — |
| CV | Coefficient of variation across C1–C6 | Float | — |
| Slope | Adaptation slope | Float | — |
| Brace_treatment | History of brace treatment | String | Yes / No |
| Physiotherapy | History of physiotherapy | String | Yes / No |
| Scoliosis_exercise | History of scoliosis-specific exercise | String | Yes / No |

**Notes:** - Missing values are coded as blank cells. - All SOT equilibrium scores are the mean of three trials per condition. When a trial resulted in a fall, the equilibrium score was recorded as 0 per standard SOT convention [19,20]. - Ratio values (C2C1, C4C1, etc.) were computed from condition-level mean equilibrium scores. - PostRes = (C1_ES + C2_ES)/2 − (C5_ES + C6_ES)/2; CV = SD(C1–C6)/Mean(C1–C6); Slope = linear regression coefficient of ES on condition number (C1→C6); StrategyShift = C1 strategy score − C6 strategy score. - Data were collected between September 2025 and June 2026 at Suzhou Research Center of Medical School, Suzhou Hospital, Affiliated Hospital of Medical School, Nanjing University (Suzhou Science & Technology Town Hospital), Suzhou, China. - This dataset is provided under a Creative Commons Attribution 4.0 International License (CC BY 4.0) as Supporting Information for the published article.
