# Supplementary material for: Thoracic kyphosis is associated with somatosensory dependence and postural control in adolescent idiopathic scoliosis
Source: PLoS One. 2026 Oct 23;21(10):e0361226. doi: 10.1371/journal.pone.0361226 (PMC13693936; doi:10.1371/journal.pone.0361226)
Supplement: Supplementary file 2 [file pone.0361226.s001.zip › S4_Table_Sensitivity_Analysis.docx]

# SX Table. Sensitivity Analysis: Exclusion of Patients Aged <10 Years (N = 34)

| Metric | N = 34 (≥10 years) | N = 42 (all) D | irection |
| --- | --- | --- | --- |
| **Primary analyses** |  |  |  |
| TK vs C4/C1 (bivariate Spearman) | ρ = 0.329, p = 0.058, n = 34 | ρ = 0.416, p = 0.006, n = 42 C | onsistent |
| TK vs C4/C2 (bivariate Spearman) | ρ = 0.336, p = 0.052, n = 34 | ρ = 0.428, p = 0.005, n = 42 C | onsistent |
| TK → C4/C1 (partial \| Cobb + Age + BMI) | ρ = 0.368, **p = 0.035**, n = 33 | ρ = 0.480, p = 0.002, n = 41 | Consistent |
| TK → C4/C2 (partial \| Cobb + Age + BMI) | ρ = 0.356, **p = 0.042**, n = 33 | ρ = 0.466, p = 0.002, n = 41 | Consistent |
| **SOT condition correlations** |  |  |  |
| Cobb vs C1 | ρ = 0.563, **p = 0.001** | ρ = 0.543, p < 0.001 | Consistent |
| Cobb vs C2 | ρ = 0.671, **p < 0.001** | ρ = 0.606, p < 0.001 | Consistent |
| Cobb vs C3 | ρ = 0.570, **p < 0.001** | ρ = 0.545, p < 0.001 | Consistent |
| Cobb vs C4 | ρ = 0.270, p = 0.123 | ρ = 0.280, p = 0.073 | Consistent |
| Cobb vs C5 | ρ = 0.326, p = 0.060 | ρ = 0.340, p = 0.028 | Consistent |
| Cobb vs C6 | ρ = 0.005, p = 0.976 | ρ = 0.030, p = 0.850 | Consistent |
| TK vs C6 | ρ = 0.365, **p = 0.034** | ρ = 0.332, p = 0.032 | Consistent |
| **C4/C1 vs postural control indices** |  |  |  |
| C4/C1 vs MeanES | ρ = 0.500, **p = 0.003** | ρ = 0.453, p = 0.003 | Consistent |
| C4/C1 vs PostRes | ρ = −0.317, p = 0.068 | ρ = −0.368, p = 0.017 | Consistent |
| C4/C1 vs CV | ρ = −0.369, **p = 0.032** | ρ = −0.410, p = 0.007 | Consistent |
| C4/C1 vs Slope | ρ = 0.368, **p = 0.032** | ρ = 0.433, p = 0.004 | Consistent |
| **Tertile analysis (Kruskal-Wallis)** |  |  |  |
| C4/C1 across TK tertiles | H = 3.74, p = 0.154 | H = 8.58, p = 0.014 C | onsistent |
| PostRes across TK tertiles | H = 5.67, p = 0.059 | H = 7.92, p = 0.019 C | onsistent |
| CV across TK tertiles | H = 6.06, **p = 0.048** | H = 8.42, p = 0.015 | Consistent |
| Slope across TK tertiles | H = 6.38, **p = 0.041** | H = 9.43, p = 0.009 | Consistent |
| **Mediation (Sobel test)** |  |  |  |
| TK → C4/C1 → MeanES | z = 1.61, p = 0.108 | z = 2.49, p = 0.013 C | onsistent |

*Values are Spearman ρ unless otherwise indicated. Bold = p < 0.05. N = 34 for the sensitivity cohort (≥10 years with complete TK data); N = 42 for the full cohort. Sample sizes for individual analyses vary due to missing data (1 additional patient missing BMI in the partial correlation models). The bivariate TK–C4/C1 correlation was attenuated (p = 0.058) but the partial correlation remained significant after covariate adjustment (p = 0.035). The complementary pattern of Cobb–firm-surface and TK–C6 associations was preserved. The tertile and mediation analyses lost statistical significance due to reduced sample size, but the direction and magnitude of associations remained consistent with the primary analysis. These results indicate that the primary findings are not driven by the inclusion of juvenile-aged patients.*
