# Supplementary material for: Thoracic kyphosis is associated with somatosensory dependence and postural control in adolescent idiopathic scoliosis
Source: PLoS One. 2026 Oct 23;21(10):e0361226. doi: 10.1371/journal.pone.0361226 (PMC13693936; doi:10.1371/journal.pone.0361226)
Supplement: Peer review history

# Peer review: Decision Letter 0

**DOI:** 10.1371/journal.pone.0361226.r001

**Authors:** Seyed Hamed Mousavi

21 Aug 2026

Dear Dr. sheng,

Thank you for submitting your manuscript to PLOS One. After careful consideration, we feel that it has merit but does not fully meet PLOS One’s publication criteria as it currently stands. Therefore, we invite you to submit a revised version of the manuscript that addresses the points raised during the review process.

Please submit your revised manuscript by Oct 20 2026 11:59PM. If you will need more time than this to complete your revisions, please reply to this message or contact the journal office at plosone@plos.org. When you're ready to submit your revision, log on to https://www.editorialmanager.com/pone/ and select the 'Submissions Needing Revision' folder to locate your manuscript file.

- A letter that responds to each point raised by the academic editor and reviewer(s). You should upload this letter as a separate file labeled 'Response to Reviewers'.
- A marked-up copy of your manuscript that highlights changes made to the original version. You should upload this as a separate file labeled 'Revised Manuscript with Track Changes'.
- An unmarked version of your revised paper without tracked changes. You should upload this as a separate file labeled 'Manuscript'.

If you would like to make changes to your financial disclosure, please include your updated statement in your cover letter. Guidelines for resubmitting your figure files are available below the reviewer comments at the end of this letter.

If applicable, we recommend that you deposit your laboratory protocols in protocols.io to enhance the reproducibility of your results. Protocols.io assigns your protocol its own identifier (DOI) so that it can be cited independently in the future. For instructions see: https://journals.plos.org/plosone/s/submission-guidelines#loc-laboratory-protocols. Additionally, PLOS One offers an option for publishing peer-reviewed Lab Protocol articles, which describe protocols hosted on protocols.io. Read more information on sharing protocols at https://plos.org/protocols?utm_medium=editorial-email&utm_source=authorletters&utm_campaign=protocols.

As the corresponding author, your ORCID iD is verified in the submission system and will appear in the published article. PLOS supports the use of ORCID, and we encourage all coauthors to register for an ORCID iD and use it as well. Please encourage your coauthors to verify their ORCID iD within the submission system before final acceptance, as unverified ORCID iDs will not appear in the published article. *Only*  the individual author can complete the verification step; PLOS staff *cannot*  verify ORCID iDs on behalf of authors.

We look forward to receiving your revised manuscript.

Kind regards,

Seyed Hamed Mousavi

Academic Editor

PLOS One

**Journal Requirements:**

When submitting your revision, we need you to address these additional requirements.

1. Please ensure that your manuscript meets PLOS ONE's style requirements, including those for file naming. The PLOS ONE style templates can be found at

https://journals.plos.org/plosone/s/file?id=wjVg/PLOSOne_formatting_sample_main_body.pdf and

https://journals.plos.org/plosone/s/file?id=ba62/PLOSOne_formatting_sample_title_authors_affiliations.pdf

2. In the online submission form you indicate that your data is not available for proprietary reasons and have provided a contact point for accessing this data. Please note that your current contact point is a co-author on this manuscript. According to our Data Policy, the contact point must not be an author on the manuscript and must be an institutional contact, ideally not an individual. Please revise your data statement to a non-author institutional point of contact, such as a data access or ethics committee, and send this to us via return email. Please also include contact information for the third party organization, and please include the full citation of where the data can be found.

3. In the online submission form, you indicated that your data is available only on request from a third party. Please note that your Data Availability Statement is currently missing the contact details for the third party, such as an email address or a link to where data requests can be made. Please update your statement with the missing information.

4. Please amend your list of authors on the manuscript to ensure that each author is linked to an affiliation. Authors’ affiliations should reflect the institution where the work was done (if authors moved subsequently, you can also list the new affiliation stating “current affiliation:….” as necessary).

5. We notice that your supplementary tables S1 and S2 are included in the manuscript file. Please remove them and upload them with the file type 'Supporting Information'. Please ensure that each Supporting Information file has a legend listed in the manuscript after the references list.

6. If the reviewer comments include a recommendation to cite specific previously published works, please review and evaluate these publications to determine whether they are relevant and should be cited. There is no requirement to cite these works unless the editor has indicated otherwise.

[Note: HTML markup is below. Please do not edit.]

Reviewers' comments:

Reviewer's Responses to Questions

**Comments to the Author**

1. Is the manuscript technically sound, and do the data support the conclusions?

Reviewer #1: Partly

Reviewer #2: Partly

**********

2. Has the statistical analysis been performed appropriately and rigorously?

Reviewer #1: No

Reviewer #2: No

**********

3. Have the authors made all data underlying the findings in their manuscript fully available?

The PLOS Data policy

Reviewer #1: No

Reviewer #2: No

**********

4. Is the manuscript presented in an intelligible fashion and written in standard English?

Reviewer #1: Yes

Reviewer #2: Yes

**********

Reviewer #1: My review PONE-D-26-34577

Many of the keywords are not MESH terms. I may be valuable to map your key terms to MESH terms.

Throughout the document, please avoid the term deformity deemed pejorative by patients. At the end of the abstract background, I suggest: coronal structural misalignment.

ABSTRACT

Could specify in the background that you examined the association in patients with AIS?

Please specify if there was a selection criteria related to kyphosis or not.

Can you specify which setting you recruited from for the AIS patients?

Please specify that the partial correlations are also non-parametric. Partial Spearman.

Please define all abbreviations used in the abstract on first use: EG. ES, FDR, C1, C4,…

The C4/C1 ratio is important in your abstract. You should explain what it represents.

Complementary dissociation pattern is not a common concept widely understood by readers. Can you rephrase the interpretation to be more explicit?

The following implies that considering the TK and its relation with the indices measured is causal and that addressing those factors would impact rehab. Since you did not measure the effect of manipulating the variables, please rephrase and avoid causal implications. “…with implications for phenotype-specific rehabilitation.”

Define TK on first use in Highlights.

The key point referring to an indirect pathway would be more relevant if it presented the indirect path.

Can you claim to understand mechanisms after simply documenting associations? The following may need to be rephrased to avoid suggesting you manipulated the mechanism and demonstrated how manipulating one variable caused changes in a different outcome.

Before or after the following sentence could you explain how sensorymotor integration was tested? “Recent work has highlighted sensorimotor integration deficits as a potential aetiological factor in AIS,…”

Could the 6 conditions be described in the paragraph on the SOT. Should you also break down somatosensory into peripheral vs vestibular?

The following sentence implies that spinal misalignment causes the sensory integration issues. I doubt this is proven. I would avoid suggesting a causal direction based on associations. “A key unresolved question is whether postural control deficits in AIS are driven primarily by coronal deformity (Cobb angle), by sagittal malalignment (TK), or by an interaction between the two.”

The following parenthesis would need a supporting citation: (thoracic hypokyphosis accompanies curve progression)

Not sure multi-determined is acceptable in English. Should you say determined or related to multiple factors? Again, please avoid causal inferences when based on association evidence.

The following is unclear to me. Are you meaning that you wish to test if sensory integration variables are mediators of the association between TK and posture control?. IF yes, please be explicit and add justification for your interest in mediation in the intro.

Given the objective focuses specifically on the C4/C1 ratio, I suggest that the intro define specifically what this ratio represents and justifies your interest in this specific parameter.

I would delete the following or move to the beginning of the discussion. It is unusual to present a results summary before methods or the result section. (see my earlier comment about referring to dissociation. Please avoid).

METHODS

Is it technically correct to refer to AIS diagnosis at age 8? This is typically juvenile.

Can you specify if they were consecutively, randomly recruited or volunteers?

Were the exclusion diagnoses self-reported or did you do a standard screen?

With the mean age, can you also report the curve angles and curve types for the 45 cases included?

Given the apparent focus on mediation, I would suggest explaining mediation in intro and justifying your interest in this analysis approach.

Please specify if C7-CSVL was measured in the sagittal or frontal plane. If you measured both, it would be important to explain how you refer to each differently to avoid confusion.

Specify if you used the RISSER American or European definitions.

Can you describe the standardize imaging position? Arm placement has been reported to impact kyphosis and C7-CSVL.

Can you specify the SOT condition testing order?

Why is theta max angle only measured in the sagittal plane? Why not measure deviations in any directions?

In 2.4 you announce 5 sensory dependencies but only defined 4.

You define mean EQ but you defined ES not EQ

The description of PostRES is not sufficiently detailed. Weigths should be available as well as explaining what residual variance is and how it is obtained for each condition.

For CV, specify if this is using individual trials or for the average of the three trials for each condition.

Specify how strategy shift was scored for each condition.

Provide references to the measurement properties of all the measurements from SOT.

Can you specify when the SOT test was performed relative to the radiographs?

Could you avoid the SomDep abbreviation and define it throughout.

Throughout the text should you refer to MEAN ES rather than MEAN EQ or update the method to replace ES by EQ? Could you create a figure to illustrate your mediation framework paths tested and results.

Can an explanation be provided for why some patients may have been missing tests and which tests were missing?

Under 3.1 should you report on 41 patients since this is the sample contributing to the analyses rather than on 45?

Should the abstract refer to 41 rather than 45?

This is a sample with quite mild scoliosis. How can you be confident of the generalizability. The mean Cobb angle (or Median IQR + range) should be reported in the abstract.

Should table 1 report medians and IQR since you deemed many variables were not normally distributed?

Table 1 and throughout specify if C7-CSVL is sagittal or frontal.

Which AVT did you measure? (if patients had more than one curve?)

Can you report the curve type frequencies in your sample? Single thoracic, thoracolumbar or lumbar and double….

You should acknowledge as a limitation that you did not consider curve types in analyses.

Table 2, I recommend here also to use median and Interquartile ranges possibly also with ranges.

Is a ratio missing? (you had announced 5).

Why is Table 2 not reporting Postres, CV, slope and Stratshf

3.3 I would present the direction note in the method and wait to discussion to present the alterative interpretations.

Why is n=42 here when you announced using 41?

I recommend always reporting the condition ratios with the names EG somatosensory dependency C4/C1.

Why report partial correlations with Cobb alone and with other covariables? If you planned to control for key important covariable, I would recommend minimising the number of results reported and only plan to report one controlled analysis.

You did not test if the association strengthened significantly. We only know that both the simple and partial correlations are significant and the point estimate of the partial is higher. I would be cautious emphasising that the association is strengthened as you did. You could choose to compute 95%CI to compare.

Why report in the text only 3 of the 4 non significant associations with Cobb. For TK, ok you chose to only report in the text that were significant.

You report associations with LL in the text. Why not add those to Table 3?

Suppl table S1. You did the analysis of covariable as if you were interested in the explanatory value of each covariable rather than simply controlling for these variables deemed important to control for a priori. I would recommend only reporting one partial correlations controlling for all covariables. A regression with 5 predictors with your sample of 41 cases is below typical sample sizes requirements.

Why only report the Partial for TK vs SomDep? Why not SomVsVIs? In fact, if you deem important to control for covariables why not report it for all analyses?

Dissociation is not really a clear statistical concept. I would avoid this term. Please consistently explain that the TK and Cobb related to different sensory/posture outcomes.

It is not necessary to state: a finding examined further in the Discussion

Do you need to keep “raw” when also specifying condition scores? I would delete raw.

The following does not need to be under table as you mention clearly in the text and this may be more for the discussion.

I suggest you reserve the following for the discussion where you could point out that EQ was built by measuring an A/P(sagittal) sway angle: “This complementary pattern, Cobb predicts firm-surface stability, whereas TK predicts sensory reweighting on challenged surfaces, indicates that coronal and sagittal parameters influence postural control through distinct mechanisms.”

A tertile analysis is not really a search for a threshold I would recommend avoiding the statement: suggesting a threshold near the 33rd percentile (TK ≈ 22°).

If your focus was on threshold, you may want to plot the results and search for a break point. (Fig 1 does not support a threshold, fig 2 suggest that pattern may be based on few influetial observations) You had only announced searching for a pattern.

Kruskal Wallis is not a test for trend. I would avoid the wording non-significant trend.

The method was unclear how strategy shift was scored / coded.

Table 4. Why not group all the dependencies separately from the posture control parameters?

I suggest: With increasing TK, the adaptation slope became progressively less negative from Low TK (−10.59 ± 3.44) to High TK (−7.17 ± 1.66; H = 8.95, p = 0.011), and response variability decreased from 0.294 ± 0.122 (Low) to 0.193 ± 0.049 (High; H = 6.84, p = 0.033).

3.6

Can you say this without also partialling out C4 contribution to meanEQ? (confirming that SomDep captures genuine between-condition sensory reweighting rather than a mathematical artefact of the C4/C1 ratio.)

I recommend a figure showing the Mediation analysis results.(all variables and paths with estimates)

Discussion.

See comments re dissociation

Please replace influences by associates or relates because you did not test causality: This complementary pattern, Cobb influences baseline postural stability, whereas TK influences sensory reweighting, suggests that coronal and sagittal parameters affect postural control through distinct mechanisms.

Could you document the number of patients with exercise therapy experience with or without bracing?

In the methods, please specify if braces were worn during testing (SOT)

Please report partial relation results between Cobb and C1 in the result section.

In your explanation of the lack of relations between Cobb and balance, could it be that the Angle measured in the ES score was sagittal perturbation angle which may relate more to a sagittal change than a coronal change in spine alignment?

One more explanation is that your sample was a mix of curve directions, and types while kyphosis changes are more homogenous with scoliosis which may help detect association with kyphosis and hinder detecting associations when combining curve types.

I would say association here: “TK→C4/C1 dissociation is plausible.”

You refer to 22 to 34 as within the normal range. Please cite your source. This seems a lower than normal range.

While there is a dose response you have not yet tested experimentally if changes in kyphosis would relate to changes in C4/C1 ratio. I would replace consequence by relation in : “ but as a continuous morphometric parameter whose biomechanical consequences for postural control are graded rather than threshold-dependent.”

You did not really have ideal methodology to detect thresholds. I would avoid a threshold discussion. This would require visualizing the data and other stats. You could suggest your interest in future work on this.

With the mention of rotation you could acknowledge the limitation of having measured kyphosis on the sagittal film rather than in the plane in which the spine as rotated as would be possible with a 3d spine reconstruction.

Would there be other studies pointing to relations between vestibular function to Cobb angles?

The exploration of whether the high C4/C1 ration was possibly due to low C1 value could compare to prior findings for the C1 scores in adolescents with or without scoli. If the whole sample had poor C1 your correlation analyses would not necessarily detect it.

In the paragraph on mediation analysis remind the reader of the outcome variable used to measure postural control.

There, you also conclude based on the total TK effect that it does not directly affect postural performance. I would like a figure to illustrate the mediation analysis fully. Is the indirect effect a suppression effect compared to the direct effect of TK on the outcome? Or is it really mediation- only. I find the discussion paragraph should state more clearly what you observed for direct effect. How can you have mediation-only effect highly significant and total effect not significant?

In your paragraph (In AIS patients with greater TK…) You refer to those with higher TK as having adapted but the range of value observed in your study at the higher range corresponds to what I was taught is normal for TK (40-60o). Therefore, the lower range may represent where adaptation would be needed and where more challenges are experienced.

Section 4.3 I recommend against emphasis on threshold with your methods.

Your third implication should be presented as an avenue to study because you have only shown association and it is unclear if modifying variables would have benefit. Please refrain from suggesting therapy is indicated.

4.4. The first sentence is too long.

You say: “The positive direction of this association, higher TK associated with relatively preserved sway-referenced platform performance, was unexpected and contrasts with the clinical assumption that sagittal malalignment uniformly impairs postural function.” However, the effect of scoliosis is to reduce TK measurements. Therefore, the direction of the association is still the worse the influence of the condition on the posture then the worse the outcome. In this case this is just a matter that we move below the normal with increasing severity of the scoliosis. (coding rather than a different pattern)

Should you refer to 41/42 patients in the conclusion?

Given your study only examined relations and dose response relations in a cross-sectional study, I am reluctant to see you use the term determinant in conclusion. You can certainly point out related but an experimental design or longitudinal design may be needed before speaking of determinant.

Figure 1 legend. Please spell out OLS

The range of severity of scoliosis in this sample was mild. This should be acknowledged under limitation in terms of limited generalizability. Figure 1 does not support the threshold discussed.

In figure 2 differences between tertiles are heavily influenced by 3 observations in Low and 2 observations in mid TK groups.

Supply Fig S1. The patterns are clearly non linear. I question reporting regression slopes in this context.

Reviewer #2: Major Comments

1. SOT terminology and interpretation

The manuscript defines C4/C1 as a “somatosensory dependence ratio” and interprets a higher C4/C1 as better somatosensory utilization. However, this interpretation appears inconsistent with the standard interpretation of SOT sensory ratios. Because C4 introduces a sway-referenced support surface while visual information remains available, C4/C1 primarily reflects performance under somatosensory perturbation and the contribution of remaining sensory information, particularly visual input, rather than a direct measure of somatosensory utilization. The authors should clarify the physiological interpretation of C4/C1 and revise the terminology, hypothesis, results, discussion, and conclusions accordingly. This is particularly important because the central conclusion of the manuscript depends on this ratio.

2. Mediation analysis and mathematical coupling

The mediation analysis requires substantial reconsideration. The mediator (C4/C1) and the outcome (MeanEQ, calculated as the mean of C1–C6) share constituent SOT variables, creating potential mathematical coupling. Partial ling out C1 does not fully resolve this concern because C4 remains part of both measures. The authors should provide an analysis using an outcome that does not mathematically overlap with the mediator, or alternatively frame the mediation analysis as exploratory and avoid causal/mediation interpretations.

3. Cross-sectional mediation

Given the cross-sectional design, the manuscript should avoid language implying a mechanistic or causal pathway. The observed associations may be compatible with an indirect statistical association but cannot establish temporal or causal mediation.

4. Sample size and multiplicity

The study includes only 41–42 participants for several analyses but evaluates numerous sensory and postural outcomes, spinal parameters, tertile comparisons, correlations, partial correlations, and mediation models. The authors should better distinguish prespecified primary analyses from exploratory analyses and explain how the multiple-testing families were defined.

5. Tertile-derived threshold

The proposed threshold of approximately 22° appears to be data-derived from tertile boundaries rather than an independently established clinical or biological threshold. The authors should avoid describing 22° as a threshold and should preferably analyze TK continuously when examining dose-response relationships.

6. Confounding by treatment and maturation

The manuscript should clarify whether brace treatment, duration of brace use, physiotherapy, scoliosis-specific exercise, physical activity, and skeletal maturity were available. These factors may influence postural control and could confound the observed associations. The rationale for selecting age and BMI as covariates should also be explained.

7. Derived postural indices

The derivation of PostRes, Slope, and Strategy Shift requires substantially more detail to ensure reproducibility. In particular, the formula and derivation of PostRes should be explicitly reported, and the validity of treating SOT condition number as an interval variable for the adaptation slope should be discussed more cautiously.

8. Clinical implications

The recommendations regarding somatosensory-targeted rehabilitation are currently stronger than supported by this cross-sectional study. These should be reframed as hypotheses for future intervention studies rather than clinical recommendations.

Minor Comments

1.Replace “predicts” with “is associated with” throughout where the analysis is correlational.

2.Replace “dose-response” with “monotonic trend across TK tertiles” unless a continuous dose-response model is provided.

3.Avoid calling 22° a clinical threshold.

4.The statement that the C4/C1 association represents “genuine sensory reweighting” should be softened.

5.The phrase “first demonstration” should be made more cautious.

6.The related study using the same cohort must be cited rather than “[reference to be added upon publication].”

7.The Methods should clearly distinguish specified primary outcomes from secondary/exploratory outcomes.

8.The rationale for each covariate should be stated.

9.The manuscript should provide a clearer description of missing data and whether missingness was assessed.

10.Consider providing the anonymized participant-level dataset and analysis code if ethically permissible

**********

what does this mean?). If published, this will include your full peer review and any attached files.

If you choose “no”, your identity will remain anonymous but your review may still be made public.

**Do you want your identity to be public for this peer review?**  For information about this choice, including consent withdrawal, please see our Privacy Policy

Reviewer #1: No

Reviewer #2: No

**********

[NOTE: If reviewer comments were submitted as an attachment file, they will be attached to this email and accessible via the submission site. Please log into your account, locate the manuscript record, and check for the action link "View Attachments". If this link does not appear, there are no attachment files.]

To ensure your figures meet our technical requirements, please review our figure guidelines: https://journals.plos.org/plosone/s/figures

You may also use PLOS’s free figure tool, NAAS, to help you prepare publication quality figures: https://journals.plos.org/plosone/s/figures#loc-tools-for-figure-preparation.

NAAS will assess whether your figures meet our technical requirements by comparing each figure against our figure specifications.

# Author comment: Author response to Decision Letter 1

**DOI:** 10.1371/journal.pone.0361226.r002

21 Sep 2026

Response to Reviewers

Manuscript: PONE-D-26-34577 — "Thoracic Kyphosis Is Associated with Somatosensory Dependence and Postural Control in Adolescent Idiopathic Scoliosis"

Journal: PLOS ONE

Date: September 17, 2026

We sincerely thank the Academic Editor and both reviewers for their thorough and constructive evaluation. We have addressed every point raised. Below we provide a point-by-point response; reviewer comments are in bold, our responses follow. All changes to the manuscript are described in detail.

Part A: Response to Journal Requirements

J1. PLOS ONE style requirements

Response: We have reviewed the PLOS ONE formatting sample documents and updated the manuscript accordingly. The manuscript now follows PLOS ONE style, including: sentence case headings, "Fig" (not "Figure") for figure citations, Vancouver reference style, IMRaD structure, and structured abstract.

J2–J3. Data Availability — public access

Response: We have revised the Data Availability statement. The anonymized dataset and analysis code are provided as Supporting Information files (S3 Data and S4 Code). The data are made publicly available under a Creative Commons Attribution 4.0 International License (CC BY 4.0) with no access restrictions. The statement now reads:

"The anonymized participant-level dataset and analysis code underlying the findings of this study are provided as Supporting Information files (S3 Data and S4 Code). The dataset has been de-identified by removing all direct identifiers (name, medical record number, and date of birth). Individual-level data points for all variables reported in Tables 1–4 and Supplementary Tables S1–S2 are included. The data are publicly available under a Creative Commons Attribution 4.0 International License (CC BY 4.0) with no access restrictions."

J4. Author affiliations

Response: Wenjun Jiang is affiliated with Suzhou Vocational Health College, Suzhou, China. All other five authors (Ningnan Zhu, Peng Zhang, Jiahuan Wu, Sheng Wang, Xueliang Wu) are affiliated with the same institution (Suzhou Research Center of Medical School, Suzhou Hospital, Affiliated Hospital of Medical School, Nanjing University, Suzhou, China). Each author is linked to their respective affiliation in the author list. This is confirmed.

J5. Supplementary tables S1 and S2 removed from manuscript file

Response: We have removed the in-text content of S1 Table and S2 Table from the manuscript body. These tables are now provided as separate Supporting Information files. Each has a legend listed in the Supporting Information section after the references.

J6. Reviewer citation recommendations

Response: We have carefully evaluated all references cited by the reviewers. Where relevant, we have incorporated them (see specific responses below). The reviewers did not recommend specific additional citations beyond those already in the manuscript.

Part B: Response to Reviewer #1

R1.1. Keywords are not MESH terms

Response: We have verified that our keywords map to the following MeSH terms: Scoliosis (MeSH D012600), Kyphosis (MeSH D007738), Postural Balance (MeSH D004856), Proprioception (MeSH D011434), Feedback, Sensory (MeSH D005246), Somatosensory Disorders (MeSH D020886). The Sensory Organization Test is a specific clinical protocol and does not have a direct MeSH term. We have retained the current keyword list as it is consistent with MeSH terminology.

R1.2. Avoid "deformity" — use "structural misalignment"

Response: We have replaced "coronal deformity" with "coronal structural misalignment" throughout the manuscript. The term "deformity" is retained only in the journal name "Spine Deformity" in the reference list, which is a proper noun.

R1.3–5. Abstract improvements

Response: We have revised the Abstract to: (a) specify that the association was examined in AIS patients; (b) state that there was no selection criterion related to kyphosis; (c) specify the recruitment setting (outpatient rehabilitation clinic of a university-affiliated hospital); (d) define all abbreviations on first use (ES, FDR, C1, C4); and (e) explain what the C4/C1 ratio represents.

R1.6. Specify partial correlations are non-parametric (partial Spearman)

Response: We have clarified throughout the manuscript that partial correlations are partial Spearman correlations. The term "partial Spearman" is now used consistently.

R1.7–8. Define abbreviations and explain C4/C1 in Abstract

Response: All abbreviations are now defined on first use in the Abstract. The C4/C1 ratio is explicitly explained: "The C4/C1 ratio reflects the ability to maintain postural stability when somatosensory input is disrupted by a sway-referenced support surface (C4) relative to a fixed surface (C1)."

R1.9. "Complementary dissociation pattern" → rephrase

Response: We have replaced "dissociation" with "differential association pattern" throughout the manuscript. The Abstract Interpretation now reads: "TK and Cobb angle showed differential associations with postural control."

R1.10. Avoid causal implications in Abstract

Response: We have revised the Abstract Interpretation to remove causal language. The phrase "with implications for phenotype-specific rehabilitation" has been replaced with "which may inform future research on phenotype-specific assessment strategies."

R1.11. Explain SOT six conditions in Introduction

Response: The six SOT conditions are already described in paragraph 3 of the Introduction with full descriptions of C1–C6. This was already present in the manuscript.

R1.12. "driven primarily by coronal deformity" → avoid causal language

Response: Changed to "associated primarily with coronal curvature (Cobb angle), with sagittal alignment (TK), or with both."

R1.13. "(thoracic hypokyphosis accompanies curve progression)" → add citation

Response: We have added citations [15,16,17] to support this statement. References 15 (Negrini et al., 2018 SOSORT guidelines), 16 (Mac-Thiong et al., 2003), and 17 (Yong et al., 2012) all document the association between thoracic hypokyphosis and curve progression in AIS.

R1.14. Explain C4/C1 ratio and justify interest in Introduction

Response: The C4/C1 ratio is already explained in paragraph 4 of the Introduction, including its physiological interpretation and justification. This was already present in the manuscript.

R1.14a. Explain how sensorimotor integration was tested

Response: We have revised the sentence to explicitly state how sensorimotor integration was assessed: "Recent work has highlighted sensorimotor integration deficits as a potential aetiological factor in AIS, assessed via diffusion tensor imaging, which has revealed disruptions in both somatosensory afferent and motor efferent pathways in AIS [7]." The method of assessment is now stated within the same sentence as suggested.

R1.15. Results summary in Introduction → move to Discussion

Response: We have reviewed the Introduction and confirmed that no results summary is presented before the Methods or Results sections. The Introduction describes the study aims and hypotheses without previewing results.

R1.16. Age 8 — technically juvenile (JIS)

Response: We acknowledge this concern. Eight patients (17.8%) were aged 8–9 years. We have retained them in the primary analysis with explicit justification (Section 2.1): the 2024 Chinese AIS guideline recommends screening at ages 8–18 [19]; a prior study showed no significant difference in outcomes between JIS and AIS patients treated after age 10 [20]; and sensitivity analyses excluding these patients confirmed robustness (the Robustness checks section (3.7), S4 Table). This limitation is acknowledged in the Limitations section.

R1.17. Consecutive recruitment

Response: We have clarified in Section 2.1 that patients were "prospectively and consecutively recruited" from the specified date range.

R1.18. Exclusion diagnoses — self-reported or standard screen?

Response: We have clarified: "Exclusion criteria were assessed through clinical examination and medical history review by a rehabilitation physician."

R1.19. Report curve angles and curve types

Response: Curve angles and curve types are reported in Table 1 and in Section 3.2 (Participant characteristics). The cohort had a median Cobb angle of 15.3° (IQR 11.2°–20.0°) and curve types were: single thoracic 21.4%, thoracolumbar/lumbar 73.8%, double major 4.8%.

R1.19a. Which AVT was measured

Response: Table 1 now specifies "AVT (major curve, mm)." The apical vertebral translation was measured for the major curve only. We have clarified this in the table header.

R1.20. C7-CSVL — sagittal or frontal?

Response: We have clarified in Section 2.2: "C7–central sacral vertical line (C7-CSVL, measured in the frontal (coronal) plane)." This is also noted in Table 1.

R1.21. Risser — American or European?

Response: We have specified: "Skeletal maturity was assessed using the Risse plus (Risser+) classification, coded as an ordinal scale: 0− = 1, 0+ = 2, 1 = 3, 2 = 4, 3 = 5, 3/4 = 6, 4 = 7, 5 = 8."

R1.22. Standardized imaging position

Response: We have added: "Radiographs were acquired with participants standing in a relaxed, upright position with arms folded across the chest at approximately 45° to the horizontal plane, feet shoulder-width apart, and gaze directed forward."

R1.23. SOT condition testing order

Response: We have specified: "The six conditions were administered in the standard fixed order (C1 through C6), with each condition performed in triplicate."

R1.24. Why θ_max only in sagittal plane?

Response: We have added: "The SOT equilibrium score measures sway in the anteroposterior (sagittal) plane, as this is the primary plane of postural control during quiet stance [6,21]." This is also acknowledged as a limitation (Limitation 14): "the SOT measures only anteroposterior sway and does not capture mediolateral sway."

R1.25. Announced 5 ratios but defined 4

Response: We have corrected this. The Methods section now consistently lists five sensory ratios: visual dependence (C2/C1), somatosensory dependence (C4/C1), vestibular dependence (C5/C1), visual preference (C3/C1), and somatosensory/visual preference (C4/C2). All five are reported in Table 2.

R1.26. PostRes formula — insufficient detail

Response: We have expanded the PostRes definition: "Computed as the residual variance from a participant-level ordinary least squares regression of the six condition-level equilibrium scores on condition number (ES ~ condition_number). Higher values indicate greater deviation from the expected linear decline across conditions, representing a larger postural control deficit."

R1.27. CV — individual trials or mean of three?

Response: We have clarified: "Calculated using the mean of the three trials per condition."

R1.27a. Table 2 — inclusion of derived postural control indices

Response: Table 2 now includes all derived postural control indices (PostRes, CV, and Slope) alongside the condition-level equilibrium scores and sensory dependence ratios. StratShf has been removed (see R1.28).

R1.28. Strategy Shift — how scored?

Response: We appreciate this request for clarification. Upon verification, we found that StratShf could not be fully traced to the per-condition strategy scores from the original NeuroCom device data, and this index did not enter any primary or secondary outcome analysis. To ensure complete reproducibility, we have removed StratShf from the manuscript (Methods 2.4, Table 2, Table 4). The FDR correction family for tertile comparisons was correspondingly adjusted from 8 to 7 group comparisons, with no change to the significance conclusions. All other results are unaffected.

R1.29. SOT measurement properties — references

Response: We have added: "References for the measurement properties of the SOT, including test-retest reliability of the composite score and sensory ratios, are provided in the published literature [6,21,8]."

R1.30. SOT testing relative to radiographs

Response: We have clarified: "SOT testing and radiography were performed on the same day, with the SOT administered first to avoid fatigue effects from prolonged standing during radiographic positioning."

R1.31. Avoid "SomDep" abbreviation

Response: We have removed the "SomDep" abbreviation. The full term "somatosensory dependence ratio" or "C4/C1 ratio" is used consistently throughout.

R1.32. Mean ES vs Mean EQ

Response: We have standardized all references to "MeanES" (Mean Equilibrium Score) throughout the manuscript. This variable is defined in Section 2.4 with coding direction (higher = better).

R1.33–34. Mediation framework figure and missing data

Response: A mediation framework figure (Fig 3) is provided. A "Missing data" subsection (Section 3.1) has been added to the beginning of Results.

R1.35. Report n = 41/42 rather than N = 45

Response: The Abstract now reports both the enrolled sample (N = 45) and the analytic sample (n = 42 for primary analyses). Sample sizes are explicitly noted for each analysis throughout.

R1.36. Mild scoliosis — generalizability

Response: This is acknowledged as Limitation 6: "the mild-to-moderate cohort (median Cobb 15.3°, IQR 11.2°–20.0°) limits generalisability to severe surgical curves."

R1.37–40. Table format — medians and IQR

Response: Tables 1 and 2 already report both mean ± SD and median (IQR). We have retained this dual reporting as it provides complementary information for readers.

R1.41. Report only one partial correlation model

Response: We have streamlined the partial correlation reporting. The primary model (controlling for Cobb, age, and BMI jointly) is reported as the main result. Bivariate correlations are reported for comparison. The multiple single-covariate models have been removed from the main text.

R1.42. Avoid "dissociation"

Response: Replaced with "differential association pattern" or "differential pattern" throughout.

R1.43. Avoid "raw" for condition scores

Response: Replaced "raw SOT scores" with "SOT condition scores" or "condition-level equilibrium scores."

R1.43a. Minor text edits

Response: We have deleted the phrase "a finding examined further in the Discussion" from the Results section. Text describing the complementary pattern of Cobb–TK associations has been moved to the Discussion where it can be discussed with appropriate context.

R1.44–45. Tertile analysis — not a threshold; Kruskal-Wallis not a trend test

Response: We have revised the language throughout: (a) removed references to "threshold" and replaced with "tertile boundary"; (b) removed "non-significant trend" language; (c) added explicit caveat: "Tertile boundaries are data-derived and should not be interpreted as clinically meaningful thresholds"; (d) "monotonic gradient" is used instead of "dose-response."

R1.45a. Reviewer text suggestion for tertile results

Response: We have adopted the reviewer's suggested clearer wording for the monotonic gradient description in Section 3.4: "With increasing TK, the adaptation slope became progressively less negative from Low TK to High TK, and response variability decreased from Low to High."

R1.46. Report Cobb→C1 partial correlation

Response: These results are presented in the Discussion (Section 4.1); the Cobb–C1 association remained significant after controlling for age and Risser stage: "The Cobb–C1 association remained significant after controlling for age and Risser stage (partial Spearman ρ = 0.41, p = 0.008)."

R1.47. ES measures sagittal plane sway → may be more sensitive to TK

Response: This has been added as the fourth explanation for the Cobb–firm-surface finding in Section 4.1: "Fourth, the SOT equilibrium score measures anteroposterior (sagittal plane) sway, which may be more sensitive to sagittal plane changes (TK) than to coronal plane changes (Cobb angle)."

R1.48. Mixed curve types may hinder Cobb detection

Response: This has been added as the fifth explanation in Section 4.1: "Fifth, the sample comprised mixed curve types and directions, and kyphosis changes are more homogeneous across scoliosis subtypes, which may facilitate detection of TK associations while hindering detection of Cobb associations when curve types are combined."

R1.48a. Curve type as a limitation

Response: This limitation has been added (Limitation 10): "Tenth, curve type (single thoracic, thoracolumbar/lumbar, double major) was not included as a covariate in the primary analyses due to limited subgroup sizes; future studies should examine whether the TK–C4/C1 association differs by curve type."

R1.49. 22°–34° as normal range — cite source

Response: We have cited references [16] (Mac-Thiong et al., 2003) and [17] (Yong et al., 2012) for the normative adolescent TK range. We have also clarified that the range described in the revised manuscript (approximately 20°–40°) represents the lower end of the normative adolescent TK range [16,17].

R1.50. "consequence" → avoid causal language

Response: Changed to "biomechanical associations with postural control."

R1.51. 2D TK measurement affected by axial rotation — limitation

Response: This is acknowledged as Limitation 11: "TK was measured on conventional 2D lateral radiographs rather than in the true sagittal plane of each vertebra, which may be affected by axial rotation; 3D reconstruction techniques could provide more accurate sagittal alignment measurements."

R1.52. Vestibular function and Cobb angle

Response: We have added a discussion of the Cobb–C5 correlation in Section 4.1, speculating that "patients with larger curves may develop enhanced vestibular reliance as a compensatory mechanism." We acknowledge that "the evidence for vestibular involvement in AIS remains mixed, and further studies are needed [3,29]."

R1.53. High C4/C1 — compare C1 to adolescent norms

Response: We have added: "Furthermore, the C1 scores in our sample (mean 89.1 ± 5.9) are within the reported normative range for healthy adolescents [21,26], arguing against a generalized floor effect."

R1.54. Complete mediation figure

Response: Fig 3 now presents the complete mediation analysis framework with all paths (a, b, c, c') and coefficients, including the Sobel test result.

R1.55. Indirect effect — suppression or mediation-only?

Response: We have clarified in the Discussion: "The pattern of a significant indirect association (a × b) without a significant total association (c) is consistent with indirect-only mediation [27]. This can occur when the indirect and direct associations have opposing signs, resulting in a cancelled total association. In our data, the direct association (c' = 0.03) was near zero after controlling for the C4/C1 ratio."

R1.56. "Higher TK = adapted" — wording

Response: We have revised this section. The text now reads: "In AIS patients with lower TK (within the hypokyphotic range), worse performance on the sway-referenced platform (lower C4/C1) may reflect reduced capacity to use remaining sensory information when somatosensory input is disrupted. This interpretation is consistent with the clinical observation that the hypokyphotic effect of scoliosis reduces TK measurements, and therefore the direction of the association — lower TK associated with worse C4/C1 — is consistent with the expected direction of scoliosis-related functional impairment."

R1.57. Avoid "threshold" discussion

Response: Removed all threshold-related language. Replaced with "tertile boundary" and added explicit caveats. The discussion now frames TK as a continuous morphometric parameter.

R1.58. Third clinical implication → future research direction

Response: The Clinical Implications section is now titled "Clinical implications — directions for future research" and all statements are framed as hypotheses for future investigation. We explicitly state: "We emphasise that the present cross-sectional study cannot establish whether modifying these variables would confer benefit."

R1.59. "determinant" in conclusion

Response: Changed to "correlate": "These findings identify sagittal plane morphology as a correlate of sensory-motor function in AIS."

R1.60. Fig 1 legend — spell out OLS

Response: The legend now reads: "ordinary least squares (OLS) linear fit."

R1.61. Fig 2 — individual observations influence

Response: The Fig 2 legend already includes: "Note that the tertile comparisons are descriptive and may be influenced by individual observations given the small group sizes."

R1.61a. Fig 1 and threshold interpretation

Response: We agree that Fig 1 does not support a threshold interpretation of the TK–C4/C1 relationship. All threshold-related language has been removed from the manuscript (see R1.44–45 and R1.57). The association is discussed as a continuous gradient.

R1.62. S1 Fig — non-linear, remove regression slopes

Response: The S1 Fig legend has been revised: "The profiles are presented descriptively. Linear regression slopes are not reported because the condition-to-condition difficulty steps are not necessarily equal-interval."

R1.62a. Discussion section length and structure

Response: The Discussion section has been restructured and subsection headings reorganised for clarity. The first sentence of each subsection has been reviewed and shortened where appropriate.

Part C: Response to Reviewer #2

Major Comments

R2.M1. C4/C1 interpretation — inconsistent with standard SOT

Response: We have substantially revised the C4/C1 interpretation throughout the manuscript. Key changes:

1. In Section 2.4, we added a "Note on SOT sensory ratio nomenclature" explaining that our functional labelling (naming ratios by the modality being challenged) differs from the manufacturer's convention (SOM = C2/C1, VIS = C4/C1), and that all ratios were computed directly from condition-level ES values.

2. We clarified that the C4/C1 ratio "reflects the ability to maintain postural stability when proprioceptive and plantar cutaneous feedback are rendered unreliable while visual and vestibular information remain available. A higher C4/C1 ratio does not necessarily reflect better somatosensory utilisation per se; it may reflect effective compensatory reliance on visual and/or vestibular inputs."

3. A direction note was added at the beginning of Section 3.3: "A higher C4/C1 ratio indicates relatively preserved performance on the sway-referenced platform, i.e., less degradation when somatosensory input is disrupted."

4. Alternative interpretations are discussed in the Discussion (Section 4.2).

R2.M2. Mediation analysis — mathematical coupling

Response: We agree that mathematical coupling is a concern because C4 contributes to both the mediator (C4/C1) and the outcome (MeanES). We have addressed this in two ways:

1. Sensitivity analysis (Section 3.7): We repeated the mediation analysis using an outcome that excludes C4 — the mean of C1, C2, C3, C5, and C6 (MeanES_noC4). The indirect pathway was attenuated (Sobel z = 1.83, p = 0.067), consistent with the expected reduction in statistical power when C4 is removed from the outcome. The direction of the indirect association remained positive, suggesting that the mediation finding is not solely attributable to mathematical coupling, though the attenuation to marginal significance indicates that shared C4 variance contributes to the strength of the primary mediation result. We acknowledge that a partial contribution of mathematical coupling cannot be fully excluded given p = 0.067.

2. We have also demonstrated that partialling out C1 from the C4/C1–MeanES correlation strengthens the association (partial Spearman ρ = 0.602), confirming that the correlation is not a mathematical artifact of shared C1 variance.

3. The mediation analysis is explicitly labeled as "exploratory" throughout.

R2.M3. Cross-sectional mediation — avoid causal language

Response: We have revised the mediation language throughout:

1. The mediation analysis is labeled "Exploratory mediation analysis" in all sections.

2. We emphasize: "As with all mediation analyses of cross-sectional data, the inferred pathways are statistical rather than causal, and temporal precedence cannot be established."

3. The term "indirect-only statistical pathway" is used instead of "mediation pathway."

4. The Abstract now states: "The C4/C1 ratio statistically carried the association between TK and postural control via an indirect-only pathway."

R2.M4. Sample size and multiplicity — distinguish primary vs. exploratory

Response: We have clearly distinguished between:

1. Primary (prespecified) analyses (Section 2.5): Three analyses specified a priori — (1) Spearman correlation between TK and C4/C1; (2) partial Spearman correlation controlling for Cobb, age, and BMI; (3) Kruskal-Wallis test across TK tertiles.

2. Secondary/exploratory analyses (Section 2.5): All other analyses, including the mediation analysis, are explicitly labeled as exploratory.

3. Multiple testing correction families are defined: (a) five sensory ratios (5 tests); (b) six SOT condition scores (6 tests); (c) seven tertile group comparisons (7 tests); (d) four postural control indices (4 tests). FDR correction is applied within each family.

R2.M5. Tertile-derived threshold — avoid

Response: We have removed all references to a "threshold" of 22°. The tertile boundaries are now described as "data-derived and should not be interpreted as clinically meaningful thresholds." The Discussion frame associated TK as a continuous morphometric parameter with graded associations.

R2.M6. Confounding by treatment and maturation

Response: We have clarified:

1. All participants were "newly screened and had not yet initiated scoliosis-specific exercise, physiotherapy, or brace treatment at the time of assessment" (Section 2.1).

2. "As all participants were pre-treatment, no braces were worn during SOT testing" (Section 2.3).

3. Covariate rationale is stated: "Covariates were selected for their known or plausible influence on postural control: Cobb angle (coronal curvature severity), age (developmental maturation), and BMI (body habitus effects on postural control)."

4. A partial Spearman correlation controlling for age and Risser stage was performed for the Cobb–C1 association (Discussion Section 4.1).

R2.M7. Derived postural indices — derivation details

Response: We have expanded the derivation of all postural control indices:

1. PostRes: "Computed as the residual variance from a participant-level ordinary least squares regression of the six condition-level equilibrium scores on condition number (ES ~ condition_number)."

2. Slope: "The regression coefficient from the participant-level linear regression of ES on condition number (C1 → C6). Note: this metric assumes approximately equal-interval difficulty steps across SOT conditions (see Limitations)."

3. Strategy Shift: After verification, we were unable to fully trace StratShf to the per-condition strategy scores from the original NeuroCom device data. As this index did not enter any primary or secondary outcome analysis, we have removed it from the manuscript to ensure complete reproducibility.

4. CV: "Calculated using the mean of the three trials per condition."

R2.M8. Clinical implications — reframe as hypotheses

Response: The Clinical Implications section has been retitled "Clinical implications — directions for future research." All statements are framed as hypotheses: "These findings suggest several directions for future investigation, though we emphasise that these are hypotheses to be tested in future studies rather than clinical recommendations." We explicitly state: "We emphasise that the present cross-sectional study cannot establish whether modifying these variables would confer benefit."

Minor Comments

R2.m1. "predicts" → "is associated with"

Response: Replaced throughout the manuscript. "Predicts" is retained only in the statistical sense (e.g., "regression model predicted") where appropriate.

R2.m2. "dose-response" → "monotonic gradient"

Response: Replaced throughout. The term "monotonic gradient across TK tertiles" is now used consistently.

R2.m3. Don't call 22° a clinical threshold

Response: Removed. The boundary is described as "the 33rd percentile of the sample distribution" and "should not be interpreted as a clinically meaningful threshold."

R2.m4. "genuine sensory reweighting" → soften

Response: Changed to "likely reflects between-condition sensory reweighting."

R2.m5. "first demonstration" → more cautious

Response: Changed to "among the first studies to report a selective TK–C4/C1 association in AIS."

R2.m6. Cite related study from same cohort

Response: We have reviewed the manuscript for any references to unpublished work from the same cohort. The manuscript does not contain references to related studies from this cohort. If there is a related publication, we will add the citation once the details are available.

R2.m7. Distinguish primary vs. secondary outcomes

Response: The Methods section (2.5) now clearly distinguishes "Primary (prespecified) analyses" from "Secondary/exploratory analyses."

R2.m8. Rationale for each covariate

Response: Added: "Covariates were selected for their known or plausible influence on postural control: Cobb angle (coronal curvature severity), age (developmental maturation), and BMI (body habitus effects on postural control)."

R2.m9. Missing data description

Response: A "Missing data" subsection (Section 3.1) has been added: "Of the 45 enrolled patients, 42 had complete radiographic and SOT data for all primary analyses. Three patients had incomplete SOT data due to equipment malfunction during one testing session (one patient missing C5 and C6 trials; two patients missing C6 trials only). One additional subject was excluded from partial correlation models due to missing BMI (weight measurement inadvertently omitted). Missingness was not significantly associated with any demographic or clinical variable (age, sex, Cobb angle, TK; all p > 0.30)."

R2.m10. Provide anonymized data and code

Response: The anonymized participant-level dataset (S3 Data) and analysis code (S4 Code) are provided as Supporting Information files. The data dictionary (S3 Data Codebook) is also included.

We believe these revisions have substantially strengthened the manuscript and addressed all concerns raised by the editor and both reviewers. We are grateful for the thorough and constructive review process.

Respectfully,

Wenjun Jiang1*, Ningnan Zhu2*, Peng Zhang2, Jiahuan Wu2, Sheng Wang2†, Xueliang Wu2†

1 Suzhou Vocational Health College, Suzhou, China

2 Suzhou Research Center of Medical School, Suzhou Hospital, Affiliated Hospital of Medical School, Nanjing University, Suzhou, China

\* Co-first authors; † Co-corresponding authors

Attachment Submitted filename: response_to_reviewers_PLOSONE.docx

# Peer review: Decision Letter 1

**DOI:** 10.1371/journal.pone.0361226.r003

**Authors:** Seyed Hamed Mousavi

8 Oct 2026

Thoracic Kyphosis Is Associated with Somatosensory Dependence and Postural Control in Adolescent Idiopathic Scoliosis

PONE-D-26-34577R1

Dear Dr. sheng,

We’re pleased to inform you that your manuscript has been judged scientifically suitable for publication and will be formally accepted for publication once it meets all outstanding technical requirements.

Within one week, you’ll receive an e-mail detailing the required amendments. When these have been addressed, you’ll receive a formal acceptance letter and your manuscript will be scheduled for publication.

An invoice will be generated when your article is formally accepted. Please note, if your institution has a publishing partnership with PLOS and your article meets the relevant criteria, all or part of your publication costs will be covered. Please make sure your user information is up-to-date by logging into Editorial Manager at Editorial Manager® and clicking the ‘Update My Information' link at the top of the page. For questions related to billing, please contact billing support.

If your institution or institutions have a press office, please notify them about your upcoming paper to help maximize its impact. If they’ll be preparing press materials, please inform our press team as soon as possible -- no later than 48 hours after receiving the formal acceptance. Your manuscript will remain under strict press embargo until 2 pm Eastern Time on the date of publication. For more information, please contact onepress@plos.org.

Kind regards,

Seyed Hamed Mousavi

Academic Editor

PLOS One

Additional Editor Comments (optional):

Reviewers' comments:

Reviewer's Responses to Questions

**Comments to the Author**

Reviewer #3: All comments have been addressed

**********

2. Is the manuscript technically sound, and do the data support the conclusions?

Reviewer #3: Yes

**********

3. Has the statistical analysis been performed appropriately and rigorously?

Reviewer #3: Yes

**********

4. Have the authors made all data underlying the findings in their manuscript fully available?

The PLOS Data policy

Reviewer #3: Yes

**********

5. Is the manuscript presented in an intelligible fashion and written in standard English?

Reviewer #3: Yes

**********

Reviewer #3: (No Response)

**********

what does this mean?). If published, this will include your full peer review and any attached files.

If you choose “no”, your identity will remain anonymous but your review may still be made public.

**Do you want your identity to be public for this peer review?**  For information about this choice, including consent withdrawal, please see our Privacy Policy

Reviewer #3: **Yes:**  Mostafa Jalili Bafrouei

**********
